# Molecular and Genetic Determinants of Nephrocalcinosis: Mechanisms, Genotype–Phenotype Correlations, and Precision Medicine

**DOI:** 10.3390/ijms27083616

**Published:** 2026-04-18

**Authors:** Setalia Popa, Andrei Cristian Grădinaru, Elena Emanuela Braha, Mihaela Grămescu, Ramona Babici, Cristina Ailenei, Lăcrămioara Ionela Butnariu

**Affiliations:** 1 Department of Medical Genetics, Faculty of Medicine, Grigore T. Popa University of Medicine and Pharmacy, 16 University Street, 700115 Iași, Romania; setalia.popa@umfiasi.ro (S.P.); gramescu.mihaela@umfiasi.ro (M.G.); ramona.babici@gmail.com (R.B.); ionela.butnariu@umfiasi.ro (L.I.B.); 2 Clinica Sante, C3 Ion Baiesu Street, 120072 Buzau, Romania; cristina.ailenei@clinica-sante.ro; 3 “Ion Ionescu de la Brad” Iasi University of Life Sciences, 3 Sadoveanu Alley, 700490 Iași, Romania; 4 “C.I. Parhon” National Institute of Endocrinology, 34–36 Aviatorilor Blvd, Sector 1, 011863 Bucharest, Romania; elena.braha@parhon.ro

**Keywords:** nephrocalcinosis, monogenic kidney disorders, tubular transport defects, mineral metabolism, genotype–phenotype correlations, precision medicine

## Abstract

Nephrocalcinosis, defined as the deposition of calcium salts within the renal parenchyma, represents a radiologic and pathologic endpoint shared by a broad spectrum of metabolic and monogenic disorders. Advances in genomic medicine have identified more than 30 genes involved in tubular transport, mineral and acid–base homeostasis, oxalate metabolism, mitochondrial function, ciliary signaling, and nephron development, reframing nephrocalcinosis as a heterogeneous manifestation of discrete molecular defects rather than a single disease entity. Despite this diversity, these conditions converge on common physicochemical pathways of tubular supersaturation, crystal nucleation, growth, and intrarenal retention. These processes are amplified by the intrinsic vulnerability of the renal medulla—characterized by hyperosmolality, hypoxia, and slow tubular flow—and by epithelial injury, loss of crystallization inhibitors, and impaired ciliary signaling. Distinct genotype–phenotype signatures, including age at onset, biochemical profiles, and extrarenal manifestations, provide important diagnostic clues and help differentiate major monogenic entities. The increasing availability of targeted gene panels, whole-exome sequencing, and whole-genome sequencing has substantially improved diagnostic yield, particularly in pediatric populations. Molecular diagnosis now directly informs therapeutic decision-making and long-term management, enabling a shift toward precision nephrology. This narrative review integrates genetic, mechanistic, and clinical perspectives to illustrate how molecular diagnosis reshapes the evaluation, prognosis, and treatment of nephrocalcinosis.

## 1. Introduction

Nephrocalcinosis (NC) refers to the deposition of calcium phosphate or calcium oxalate within the renal cortex or medulla and represents a radiologic and histopathologic finding rather than a single disease entity, a distinction emphasized in major reviews of inherited and metabolic stone disease [1,2]. It arises from diverse metabolic and genetic disturbances and is particularly prevalent in neonates, infants, and children, in whom monogenic causes are disproportionately represented [3,4].

Although often detected incidentally, nephrocalcinosis may reflect underlying disorders associated with significant long-term renal morbidity, including progression to chronic kidney disease.

Over the past decade, advances in genomic medicine have fundamentally reshaped the diagnostic landscape of nephrocalcinosis. More than 30 genes have been implicated in monogenic forms of the disease [1,5], affecting pathways such as tubular transport, mineral and acid–base homeostasis, oxalate metabolism, mitochondrial function, ciliary signaling, and nephron development [6,7,8,9,10]. The increasing use of next-generation sequencing (NGS), including targeted gene panels, whole-exome sequencing (WES), and whole-genome sequencing (WGS), has significantly improved diagnostic yield [4,11], particularly in pediatric populations, where detection rates of 20–40% are routinely reported and may be even higher in consanguineous populations [7,9]. These advances have reframed nephrocalcinosis as a convergent phenotype arising from distinct molecular defects rather than a uniform clinical entity.

Despite this genetic and biochemical heterogeneity, the underlying pathogenic processes converge on a limited number of shared physicochemical and biological mechanisms, including tubular supersaturation, crystal nucleation, growth, impaired clearance, and progressive intrarenal retention [6,8,10]. These processes are further amplified by the intrinsic vulnerability of the renal medulla, characterized by hyperosmolality, hypoxia, and low tubular flow, as well as by epithelial injury and loss of endogenous crystallization inhibitors. Importantly, distinct genotype–phenotype correlations, including age at onset, biochemical signatures, and extrarenal manifestations, provide critical diagnostic clues and guide clinical evaluation.

The clinical implications of molecular diagnosis extend beyond etiological clarification. Identification of the underlying genetic defect informs prognosis, enables targeted therapeutic strategies, supports family counseling, and increasingly guides precision medicine approaches. Examples include RNA interference therapies for primary hyperoxaluria [12,13], alkali therapy in distal renal tubular acidosis [14,15], and magnesium supplementation for familial hypomagnesemia with hypercalciuria and nephrocalcinosis [16,17]. However, significant challenges remain, including incomplete genotype–phenotype correlations, variable penetrance, and the complex interplay between genetic defects and the renal microenvironment [4,11].

In this context, the present narrative review aims to integrate molecular, pathophysiological, and clinical perspectives on nephrocalcinosis. The manuscript is structured to progress from the biological mechanisms underlying crystal formation, through the genetic architecture of monogenic disease, to genotype–phenotype correlations, diagnostic strategies, and mechanism-directed therapeutic approaches. Particular emphasis is placed on pediatric nephrocalcinosis, where early recognition of monogenic etiologies has the greatest potential to alter clinical outcomes [3,18].

## 2. Epidemiology and Clinical Relevance of Nephrocalcinosis

Early diagnosis of nephrocalcinosis is of critical clinical importance, as it enables timely identification of the underlying etiology and the initiation of mechanism-directed interventions aimed at preventing irreversible renal damage [19]. Although often detected incidentally on imaging, nephrocalcinosis may represent the earliest manifestation of metabolic or monogenic disorders with significant long-term consequences, including progressive chronic kidney disease [19].

Despite its clinical relevance, the epidemiology of nephrocalcinosis remains insufficiently defined. Population-level prevalence data are lacking, largely due to heterogeneity in diagnostic criteria, study populations, and imaging practices [19]. Although reported detection rates have increased substantially over recent decades, this trend primarily reflects improved ascertainment driven by the widespread use of renal ultrasonography and cross-sectional imaging, as well as increased clinical awareness and targeted screening in at-risk groups, rather than a well-characterized change in true disease prevalence [20]. Importantly, most available data derive from selected cohorts, including tertiary referral populations and neonatal screening programs, rather than from population-based studies [19]. Consequently, the true burden of nephrocalcinosis remains uncertain and is likely underestimated in administrative registries and epidemiological databases [19]. In this context, epidemiological data on nephrolithiasis (a related but distinct phenotype affecting approximately 10% of adults in many high-income countries) provide only indirect insight, further underscoring the under-recognition of renal parenchymal calcification [21].

The epidemiological profile of nephrocalcinosis varies significantly across age groups [21]. In adults, the condition is most commonly associated with acquired metabolic disturbances, such as hypercalciuria, hyperparathyroidism, or chronic kidney disease [20]. In contrast, pediatric nephrocalcinosis is characterized by a distinct etiological profile, with a disproportionately high contribution of monogenic disorders affecting tubular transport, mineral metabolism, and oxalate handling [19]. Genetic causes are identified in approximately 20–40% of unselected pediatric cohorts and in up to 70% of selected populations, particularly in early-onset or familial cases [3].

A special clinical context is represented by preterm infants, in whom nephrocalcinosis is frequently detected during routine ultrasound screening, with reported incidences ranging from 7% to over 40% depending on gestational age and cohort characteristics [22]. In many of these cases, nephrocalcinosis is transient and resolves spontaneously within the first months of life [22]. However, distinguishing these benign, prematurity-associated forms from genetically determined nephrocalcinosis is essential, as the latter are associated with persistent disease, early renal impairment, and specific therapeutic implications [23].

From a clinical perspective, nephrocalcinosis should not be regarded as a uniform condition but rather as a heterogeneous phenotype with variable prognostic significance. While some cases remain asymptomatic or self-limited, others reflect severe underlying disorders with a high risk of progression to end-stage kidney disease. Importantly, molecular diagnosis has been shown to alter clinical management in 20–40% of cases, influencing therapeutic decisions, surveillance strategies, and transplant planning [3].

As summarized in Table 1, recent cohort studies and genetic analyses highlight the marked heterogeneity of pediatric nephrocalcinosis, encompassing both transient forms associated with prematurity and monogenic disorders with high diagnostic yield and significant clinical impact. This variability supports a structured, mechanism-based diagnostic approach that integrates epidemiological context with biochemical profiling and genetic testing to enable accurate diagnosis and personalized management.

## 3. Pathophysiological Basis of Nephrocalcinosis

Nephrocalcinosis arises from a complex interplay between physicochemical conditions, tubular epithelial integrity, and renal microenvironmental factors that collectively favor the formation, retention, and accumulation of calcium salts within the renal parenchyma. Although the initiating causes may vary widely, these diverse disturbances converge on a limited number of shared biological pathways that regulate crystal nucleation, growth, and intrarenal persistence [28,29]. Understanding these mechanisms provides a unifying framework linking metabolic, genetic, and environmental factors to a common pathological endpoint.

### 3.1. The Normal Medullary Microenvironment

The renal medulla represents a uniquely vulnerable anatomical and physiological compartment that predisposes to crystal formation [2,30,31]. This susceptibility is primarily determined by three interrelated features: *(i)* hyperosmolality, *(ii)* relative hypoxia, and *(iii)* reduced tubular flow. Collectively, these intrinsic features define the renal medulla as a permissive microenvironment in which relatively minor disturbances in solute handling can initiate crystal formation and retention, explaining its central role in the pathogenesis of nephrocalcinosis [1,30,32,33].

*(i)*  *The corticomedullary osmotic gradient*, generated by countercurrent multiplication and urea recycling, concentrates solutes to levels that frequently approach or exceed their solubility limits [34,35]. This hyperosmolar environment increases the ionic activity of calcium, phosphate, and oxalate, thereby promoting supersaturation even in the absence of overt systemic metabolic disturbances [32,36].
*(ii)*  In parallel, the medulla operates under *conditions of physiologically low oxygen tension*, resulting from the architecture of the *vasa recta* and the high metabolic demands of tubular transport processes [8,37]. This chronic hypoxic state limits epithelial regenerative capacity and increases susceptibility to injury under sustained physicochemical stress [38,39].
*(iii)*  *Tubular flow dynamics* further amplify this vulnerability. Flow rates progressively decline along the loop of Henle and collecting ducts, reducing the mechanical clearance of nascent crystals and prolonging their intraluminal residence time [34,40]. As a result, even transient supersaturation events may translate into persistent crystal retention [30,32].

### 3.2. Interstitial Responses and Progression to Chronic Kidney Injury

Once crystals form within the tubular lumen, their interaction with the tubular epithelium initiates a cascade of cellular and interstitial events that transform a physicochemical process into structural kidney damage [2,31].

Crystal adhesion to the epithelial surface disrupts cellular integrity and triggers local inflammatory signaling [30]. Injured epithelial cells release danger-associated molecular patterns (DAMPs), activating innate immune pathways such as the *NLRP3* inflammasome and promoting the production of pro-inflammatory cytokines [31,32]. This inflammatory response enhances epithelial stress and facilitates the recruitment of immune and mesenchymal cells into the interstitial compartment [8,37].

Sustained injury promotes epithelial–mesenchymal crosstalk, leading to fibroblast activation, myofibroblast differentiation, and extracellular matrix deposition [41]. Progressive interstitial remodeling distorts tubular architecture, impairs microcirculation, and further disrupts solute handling [42].

In parallel, repeated cycles of epithelial injury and incomplete repair result in tubular atrophy, basement membrane thickening, and expansion of the interstitial space [2,30]. These structural changes reduce tubular flow and enhance crystal retention, creating a self-reinforcing loop that drives the progression from microscopic crystalluria to stable intrarenal deposits [34,40].

Over time, interstitial remodeling contributes to the formation of plaque-like mineralized lesions at the corticomedullary junction, analogous to Randall’s plaques described in nephrolithiasis [32,35]. Although their composition and clinical implications vary across disorders, these lesions represent histopathological markers of chronic mineral stress and sustained tubular injury [30,43]. As fibrosis advances, nephron loss becomes irreversible, and nephrocalcinosis evolves from a radiological finding into a predictor of chronic kidney disease [33,44].

This pathogenic continuum, from crystal adhesion and inflammatory activation to fibrosis, nephron loss, and irreversible renal damage, underscores the importance of early recognition and timely, mechanism-directed intervention in metabolic and inherited disorders associated with nephrocalcinosis [18,45].

### 3.3. Core Etiopathogenic Mechanisms

Despite their diverse origins, metabolic and inherited disturbances converge on a limited set of core mechanisms that govern crystal formation and retention within the kidney [19,30,46]. These mechanisms operate at the interface between tubular fluid composition, epithelial integrity, and renal microenvironmental conditions and provide the conceptual framework for understanding nephrocalcinosis as a convergent phenotype.

#### 3.3.1. Physicochemical Determinants of Renal Crystal Formation

Several metabolic and physiological factors increase tubular supersaturation by altering the balance between solute concentration and crystallization inhibition. Hypercalciuria, hypocitraturia, and renal phosphate wasting elevate free calcium and phosphate activity or reduce citrate availability, a major endogenous inhibitor of nucleation and aggregation [30,47]. These alterations shift the physicochemical equilibrium toward precipitation, particularly in distal tubular segments where urinary concentration increases.

Disturbances in oxalate handling constitute a parallel but convergent pathway toward supersaturation. Even modest elevations in urinary oxalate sharply reduce calcium solubility, as calcium oxalate crystals remain thermodynamically stable across the physiological pH range [32,48]. As a result, increased oxalate delivery, whether due to intestinal hyperabsorption or endogenous overproduction, creates a highly lithogenic environment that predisposes to early and extensive crystal nucleation [13,49,50].

Collectively, these physicochemical perturbations shift the tubular milieu toward a state in which crystal formation becomes energetically favorable, establishing the foundation for downstream processes of crystal growth, aggregation, and intrarenal retention within the renal parenchyma [1,2,31].

#### 3.3.2. Tubular Injury and Impaired Crystal Clearance

Tubular epithelial integrity plays a central role in preventing intrarenal crystal retention. When epithelial cells are injured by metabolic stressors such as hypercalciuria or hyperoxaluria [12], local oxidative damage, or sustained physicochemical stress, the apical membrane undergoes structural and biochemical alterations that markedly enhance crystal adhesion [35]. These changes include exposure of negatively charged phospholipids, increased membrane roughness, and disruption of the glycocalyx, all of which facilitate the attachment of calcium phosphate and calcium oxalate crystals to the tubular surface. In parallel, epithelial stress reduces the expression and secretion of key crystallization inhibitors, including osteopontin and Tamm–Horsfall protein (uromodulin) [32]. Under physiological conditions, these molecules inhibit crystal aggregation and promote luminal clearance. Their depletion, therefore, removes an important protective barrier against intratubular crystal retention.

Sustained epithelial injury further activates inflammatory and fibrotic signaling pathways [19], leading to interstitial remodeling that impairs tubular flow dynamics and reduces the mechanical washout of crystals. These processes are particularly detrimental in the renal medulla, where low oxygen tension and high osmolarity favor crystal stability and persistence [35]. As a result, even modest disturbances in mineral handling can progress from transient crystalluria to fixed intrarenal deposits, underscoring tubular injury as a key amplifying factor in the pathogenesis of nephrocalcinosis. Importantly, early nephrocalcinosis may be partially reversible when tubular integrity is restored, especially in conditions where timely correction of the underlying metabolic disturbance reduces ongoing crystal deposition. This highlights epithelial injury and repair capacity as dynamic modifiers of disease progression rather than merely passive consequences of crystal formation.

#### 3.3.3. Acid–Base and Electrolyte Disturbances

Acid–base and electrolyte disturbances exert a profound influence on the physicochemical environment of the distal nephron, shaping the propensity for intratubular calcium salt precipitation. Impaired distal acidification leads to persistently alkaline urinary pH, a key driver of calcium phosphate supersaturation [51]. Alkaline urine (pH > 6.0) favors the formation of brushite and hydroxyapatite, while reduced proton availability further destabilizes tubular mineral homeostasis. Consequently, sustained elevations in urinary pH represent a major risk factor for calcium phosphate nephrocalcinosis, particularly in early life, underscoring the central role of acid–base balance in modulating crystal formation [14,52].

Disturbances in magnesium handling provide an additional pathway toward crystal deposition [53]. Magnesium acts as an endogenous inhibitor of calcium oxalate and calcium phosphate crystallization; therefore, its urinary depletion removes a critical protective factor against nucleation and aggregation, promoting both supersaturation and crystal aggregation [34,40]. Reduced magnesium availability amplifies supersaturation and promotes crystal growth, especially in the context of concomitant hypercalciuria, thereby intensifying the lithogenic milieu within the distal nephron.

Alterations in phosphate handling further modify the calcium–phosphate product and influence crystal formation. Increased urinary phosphate excretion disrupts the tightly regulated phosphate–vitamin D axis, leading to compensatory elevations in 1,25-dihydroxyvitamin D activity [47]. This hormonal imbalance enhances intestinal calcium absorption and suppresses parathyroid hormone secretion, collectively increasing the filtered calcium load and urinary calcium excretion [54]. The resulting biochemical environment is highly favorable to calcium phosphate precipitation within the distal tubular segments.

Taken together, disturbances in acid–base regulation and electrolyte homeostasis converge to destabilize the distal tubular microenvironment, lowering the threshold for calcium salt supersaturation and precipitation. These physicochemical shifts act synergistically with the medullary vulnerability described above, promoting crystal retention and progression to nephrocalcinosis even in the absence of severe systemic metabolic derangements.

#### 3.3.4. Mitochondrial and Metabolic Contributions

Mitochondrial function plays a central role in maintaining tubular homeostasis, and its disruption shapes the intrarenal environment in ways that strongly predispose crystal formation and nephrocalcinosis. Mitochondria provide the ATP required for multiple energy-dependent transport processes along the nephron, including calcium reabsorption in the thick ascending limb and proton secretion in the distal tubular segments. When oxidative phosphorylation is impaired, ATP depletion and opening of mitochondrial permeability transition pores (MPTP) lead to intracellular calcium accumulation and impaired acid–base regulation [55]. This bioenergetic failure is closely coupled with an increased generation of reactive oxygen species (ROS), particularly from the electron transport chain, which act as potent mediators of tubular injury [38]. Oxidative stress further damages epithelial cell membranes, disrupts cytoskeletal organization, and exposes adhesive molecules, including osteopontin and annexin II, thereby enhancing crystal attachment to the apical membrane [2]. These changes reduce epithelial resilience and promote intratubular crystal retention.

Metabolic disturbances that alter intracellular redox balance or organic acid metabolism converge on similar mitochondrial stress pathways. Excessive delivery of poorly soluble metabolites to the tubular lumen increases the crystallization burden and overwhelms epithelial defense mechanisms, leading to intratubular precipitation and progressive nephrocalcinosis. Mitochondrial dysfunction is further amplified by crystal-induced epithelial injury, which promotes pro-inflammatory signaling, the development of a senescence-associated secretory phenotype, and a reduction in endogenous crystallization inhibitors [12,56].

Together, mitochondrial dysfunction and metabolic stress act as powerful amplifiers of nephrocalcinosis by coupling impaired energy metabolism with oxidative injury and defective tubular transport. These processes lower the threshold for crystal formation and retention, linking disturbances in cellular energetics to progressive mineral deposition within the renal parenchyma.

#### 3.3.5. Ciliary Dysfunction and Developmental Pathways

Ciliary dysfunction disrupts essential mechanisms of tubular flow sensing, epithelial polarity, and coordinated solute transport, creating a structural and biochemical environment that strongly predisposes to nephrocalcinosis [57]. Primary cilia function as mechanosensory organelles that detect luminal shear stress, translate mechanical stimuli into intracellular signaling pathways governing epithelial differentiation, planar cell polarity, and vectorial transport [39,58]. Through these processes, cilia play a central role in maintaining tubular architecture, luminal patency, and effective solute handling along the nephron.

When ciliary structure or signaling is impaired, tubular epithelial cells lose their ability to sustain normal polarity and coordinated transport, leading to chronic alterations in tubular fluid composition and flow dynamics. These disturbances impair the reabsorption of key solutes, disrupt acid–base balance, and promote physicochemical conditions that favor supersaturation within the tubular lumen [33,37]. In parallel, disruption of flow-dependent calcium signaling further contributes to localized abnormalities in mineral handling, amplifying the lithogenic environment [59].

**Figure 1:**
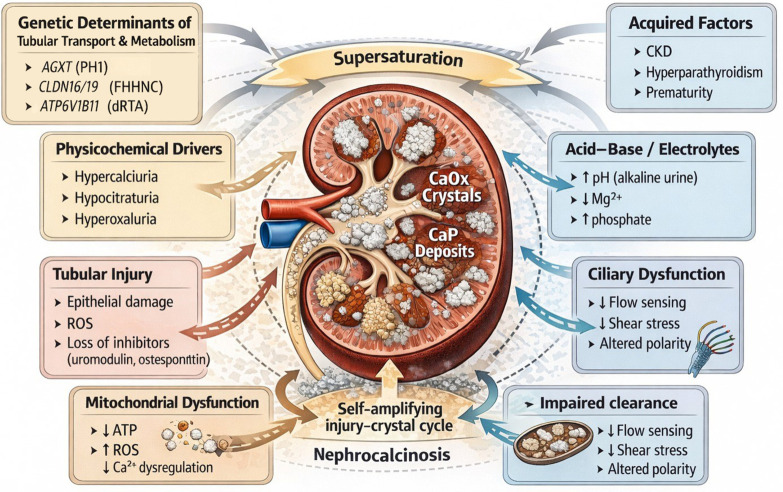
Integrated schematic of nephrocalcinosis pathogenesis. The central kidney diagram depicts intrarenal deposition of calcium oxalate and calcium phosphate crystals driven by supersaturation and a self-amplifying injury–crystal cycle. Surrounding panels summarize major contributors: genetic determinants of tubular transport and metabolism (e.g., *AGXT*, *CLDN16/19*, *ATP6V1B1*), physicochemical drivers (hypercalciuria, hypocitraturia, hyperoxaluria), tubular and mitochondrial injury (epithelial damage, ↓ATP, ↑ROS, loss of inhibitory proteins), acid–base and electrolyte disturbances (alkaline urine, ↓Mg^2+^, ↑phosphate), ciliary dysfunction and impaired clearance (reduced flow sensing and shear stress), and acquired factors (CKD, hyperparathyroidism, prematurity). This integrated model highlights how genetic, biochemical, and structural insults converge to promote crystal formation, retention, and progressive renal injury. Within the schematic, color-coded panels distinguish between systemic and physicochemical factors (tan and blue) versus localized cellular and structural dysfunctions (red and gray). Solid and dashed arrows indicate the directional influence of these diverse contributors converging on the central pathogenic mechanisms. Additionally, the circular arrangement of arrows at the base illustrates the vicious cycle of disease, wherein crystal deposition triggers further epithelial injury, which in turn promotes additional crystal retention and progressive renal damage.

Structural abnormalities associated with ciliary dysfunction distort tubular geometry, narrow the lumen, and impair the coordinated peristaltic-like movements that facilitate urine flow [39,57], as illustrated in Figure 1. The resulting reduction in shear stress creates regions of luminal stasis, a physical condition that enhances crystal adhesion and reduces the clearance of newly formed calcium phosphate or calcium oxalate particles [34,40]. Ciliary dysfunction is also frequently accompanied by tubular atrophy and interstitial fibrosis, which further slows tubular transit and exacerbates crystal retention [2,31].

Low shear environments alter crystal kinetics in a substrate-specific manner. For calcium phosphate, stagnant tubular flow permits the local accumulation of hydroxyl and phosphate ions, sustaining the alkaline microenvironment required for hydroxyapatite and brushite nucleation [32,36]. For calcium oxalate, impaired shear stress signaling induces apical expression of adhesion molecules, including hyaluronan and osteopontin, creating a highly adhesive epithelial surface [30]. In the absence of adequate flow-mediated clearance, nascent crystals remain in prolonged contact with these surfaces, facilitating their retention and aggregation [34,40].

Together, the combined failure of mechanosensory signaling, tubular architecture, and flow-dependent clearance drives the progression from transient crystalluria to fixed medullary deposits. These processes define a characteristic pathway through which ciliary and developmental disturbances promote nephrocalcinosis, independent of the specific molecular lesion responsible for ciliary dysfunction [1,33].

## 4. Genetic Architecture of Monogenic Nephrocalcinosis

Monogenic nephrocalcinosis encompasses a heterogeneous group of inherited disorders affecting key pathways involved in tubular transport, mineral metabolism, acid–base regulation, oxalate handling, mitochondrial function, and nephron development [10]. Although these conditions arise from distinct molecular defects, they converge on the core pathophysiological processes outlined in Section 3, including tubular supersaturation, impaired crystal clearance, and progressive intrarenal mineral deposition [60,61].

Organizing these disorders according to biological pathways, rather than as isolated gene lists, facilitates clinical interpretation and improves diagnostic efficiency. This pathway-oriented framework also highlights how specific genetic defects generate characteristic biochemical signatures and clinical phenotypes, thereby supporting a mechanism-based approach to diagnosis and management.

### 4.1. Disorders of Calcium and Magnesium Transport

Inherited disorders of calcium and magnesium transport represent one of the most frequent and mechanistically well-defined causes of monogenic nephrocalcinosis. These conditions disrupt tightly regulated homeostatic systems responsible for divalent cation handling along the nephron, leading to sustained hypercalciuria, impaired crystallization inhibition, and enhanced intrarenal crystal deposition [10]. As outlined in Section 3, alterations in calcium and magnesium fluxes directly modify tubular supersaturation, epithelial vulnerability, and crystal retention, providing a clear mechanistic link between genetic defects and nephrocalcinosis.

#### 4.1.1. Paracellular Transport Defects: *CLDN16* and *CLDN19*

The tight junction proteins, claudin 16 and claudin 19, form a selective paracellular channel in the thick ascending limb (TAL) of the loop of Henle, enabling coordinated reabsorption of Ca^2+^ and Mg^2+^ driven by the lumen-positive transepithelial voltage [62]. Loss-of-function mutations in either gene disrupt this pathway, resulting in renal magnesium wasting, hypercalciuria, and increased delivery of calcium to the distal nephron segments [63]. The consequent elevation in luminal calcium concentration promotes calcium phosphate supersaturation and early medullary crystal deposition.

Beyond altered divalent cation handling, dysfunction of the thick ascending limb perturbs the corticomedullary osmotic gradient, further compromising the kidney’s ability to maintain a non-lithogenic environment [64]. Magnesium depletion removes a key endogenous inhibitor of calcium salt crystallization, amplifying both nucleation and aggregation processes [34,40]. Clinically, these mechanisms manifest as familial hypomagnesemia with hypercalciuria and nephrocalcinosis (FHHNC), typically presenting in childhood with progressive medullary nephrocalcinosis and declining renal function [17]. Extra-renal manifestations, particularly severe ocular abnormalities in *CLDN19*-related disease, further distinguish this genetic subtype.

#### 4.1.2. Transcellular Calcium Sensing and Signaling Defects: *CASR*

The calcium-sensing receptor (CaSR) plays a pivotal role in regulating renal calcium handling by modulating both paracellular permeability in the thick ascending limb and transcellular calcium reabsorption in the distal convoluted tubule [65]. Activating mutations in *CASR* increase receptor sensitivity to extracellular calcium, suppressing paracellular calcium transport in the thick ascending limb and reducing *TRPV5*-mediated calcium reabsorption in the distal convoluted tubule [66]. This dual effect produces renal calcium wasting, hypercalciuria, and an increased distal calcium load, creating a high-risk environment for nephrocalcinosis despite normal or low serum calcium levels [67]. Conversely, inactivating *CASR* mutations impair calcium-dependent feedback regulation, altering tubular calcium handling in a context-dependent manner that may also predispose to crystal formation [68,69]. In both scenarios, the primary pathogenic driver is sustained by hypercalciuria coupled with reduced buffering capacity against crystallization, linking CaSR dysfunction directly to the supersaturation and retention mechanisms.

However, across paracellular and transcellular transport defects, a unifying pathophysiological theme emerges: disruption of divalent cation reabsorption increases distal calcium delivery while simultaneously reducing magnesium-mediated inhibition of crystal formation, mechanisms well documented in inherited tubulopathies and transport disorders [17,63,64]. These combined effects lower the threshold for calcium phosphate and calcium oxalate precipitation, particularly within the medullary microenvironment characterized by hyperosmolarity, hypoxia, and slow tubular flow [2,8]. As a result, disorders of calcium and magnesium transport represent a prototypical genetic entry point into the mechanistic cascade leading to nephrocalcinosis [16,70].

### 4.2. Phosphate-Wasting Disorders

Inherited defects in proximal tubular phosphate handling form a well-defined group of monogenic disorders that promote nephrocalcinosis by altering the balance between phosphate reabsorption, vitamin D metabolism, and calcium homeostasis [19]. Under physiological conditions, the proximal tubule reabsorbs the majority of filtered phosphate through the sodium-dependent cotransporters NaPi IIa and NaPi IIc encoded by *SLC34A1* and *SLC34A3*, respectively. The activity of these transporters is dynamically regulated by dietary phosphate intake, parathyroid hormone, and fibroblast growth factor 23 (*FGF23*) [71,72].

When loss-of-function mutations impair proximal tubular phosphate reclamation, urinary phosphate wasting ensues, leading to hypophosphatemia and a compensatory rise increase in 1,25 dihydroxyvitamin D synthesis. Enhanced vitamin D activity augments intestinal calcium absorption and increases the filtered calcium load, thereby promoting hypercalciuria and elevating the supersaturation of calcium phosphate in the distal nephron segments [5]. These biochemical alterations directly interface with the physicochemical determinants of crystal formation and are further amplified by the medullary vulnerability.

#### 4.2.1. *SLC34A1* and *SLC34A3*: NaPi-IIa/IIc Cotransporter Defects

Pathogenic variants in *SLC34A1* or *SLC34A3* reduce phosphate reclamation in the proximal tubule, producing a characteristic biochemical signature of hypophosphatemia, elevated 1,25(OH)_2_D levels, hypercalciuria, and nephrocalcinosis [73]. In infants, *SLC34A1* mutations may present with severe hypercalcemia and early nephrocalcinosis due to dysregulated vitamin D activation, a phenotype often classified as Idiopathic Infantile Hypercalcemia (IIH) type 2 [19,74]. In contrast, *SLC34A3* defects more commonly manifest as hereditary hypophosphatemic rickets with hypercalciuria (HHRH), characterized by skeletal deformities, bone pain, and growth impairment [75].

Despite differences in clinical presentation, both conditions share a unifying pathophysiological mechanism: increased distal calcium delivery in the setting of phosphate depletion. This biochemical milieu favors calcium phosphate precipitation, particularly when accompanied by alkaline urinary pH or reduced crystallization inhibition, thereby creating a strong lithogenic drive toward medullary nephrocalcinosis.

#### 4.2.2. *FGF23* and *GALNT3*: Abnormalities of Phosphate Regulation

Less frequently, nephrocalcinosis arises from inherited defects in systemic phosphate regulation mediated by *FGF23* or its post-translational modification enzyme *GALNT3* [76]. Pathogenic variants in these genes disrupt normal *FGF23* signaling, impairing the hormone’s ability to suppress renal phosphate reabsorption and downregulate vitamin D activation. The resulting endocrine imbalance leads to inappropriately elevated 1,25(OH)_2_D levels, hypercalciuria, and an increased calcium–phosphate product, all of which favor intratubular precipitation of calcium phosphate salts.

Clinically, these disorders may be accompanied by skeletal abnormalities, including tumoral calcinosis or rickets, depending on the direction and magnitude of *FGF23* dysregulation and its interaction with the klotho–FGF receptor complex [77,78]. Renal mineral deposition reflects the same physicochemical principles described for proximal tubular transporter defects, reinforcing the central role of the phosphate–vitamin D–calcium axis in the pathogenesis of nephrocalcinosis.

However, across phosphate-wasting disorders, a consistent mechanistic theme emerges: disruption of phosphate homeostasis leads to maladaptive activation of vitamin D pathways, increased intestinal calcium absorption, and sustained hypercalciuria, mechanisms that are well described in endocrine and renal physiology studies [47,79,80]. These changes amplify calcium phosphate supersaturation within the distal nephron and intersect with the medullary microenvironmental vulnerabilities outlined in Section 3, culminating in progressive nephrocalcinosis [2,8]. Consequently, inherited phosphate-wasting syndromes represent a key genetic category linking endocrine dysregulation to renal crystal deposition, as demonstrated in disorders caused by *SLC34A1* and *SLC34A3* mutations [54,75].

### 4.3. Acid–Base Regulation Disorders

Disturbances in distal acidification represent a central genetic mechanism through which several monogenic disorders promote nephrocalcinosis. Maintenance of an acidic urinary environment depends on tightly coordinated proton secretion and bicarbonate reclamation in the distal nephron. When these processes are disrupted, urinary pH rises into a range that markedly favors calcium phosphate supersaturation, particularly the formation of brushite and hydroxyapatite [19,81]. Persistently alkaline urine also reduces the solubility of phosphate and diminishes the activity of endogenous crystallization inhibitors, thereby creating a permissive milieu for intratubular precipitation [39].

Pathogenic variants in *ATP6V1B1* and *ATP6V0A4*, encoding key subunits of the vacuolar H^+^-ATPase, impair proton secretion in α-intercalated cells of the collecting duct and lead to distal renal tubular acidosis (dRTA) [82]. The resulting metabolic acidosis is characteristically accompanied by hypercalciuria, hypocitraturia, and alkaline urine [74], three synergistic drivers of calcium phosphate crystal formation. In affected children, nephrocalcinosis often develops early and may progress despite adequate correction of systemic acidosis, underscoring the dominant role of urinary pH in the medullary microenvironment [15].

Defects in *SLC4A1*, which encodes the basolateral Cl^−^/HCO_3_^−^ exchanger (AE1), produce a similar pathogenic cascade by impairing bicarbonate efflux from intercalated cells. This disturbance disrupts intracellular acid–base balance, reduces proton availability for luminal secretion, and results in incomplete or complete forms of dRTA [73]. As in H^+^-ATPase–related disease, the combination of alkaline urine and increased distal calcium delivery promotes calcium phosphate precipitation along the collecting ducts [5].

Across acid–base regulation disorders, a unifying pathophysiological theme emerges: failure to maintain an appropriately acidic urinary milieu shifts the physicochemical balance toward supersaturation, enhances crystal nucleation, and accelerates medullary mineral deposition. By directly modifying urinary pH and crystallization inhibition, acid–base defects constitute a major and highly penetrant genetic pathway leading to monogenic nephrocalcinosis [7,19].

**Figure 2:**
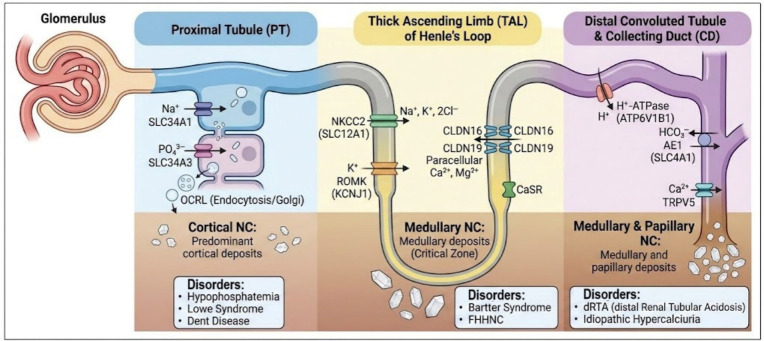
The Genetic Map of Nephrocalcinosis: Segmental distribution of transporters and genetic defects leading to nephrocalcinosis. The proximal tubule is the primary site for phosphate reabsorption (*SLC34A1/3*) and endocytosis (*OCRL*). The Thick Ascending Limb (TAL) facilitates paracellular transport of Ca^2+^ and Mg^2+^ via Claudins 16 and 19, regulated by the CaSR. Distal segments are crucial for acid-base balance through H^+^-ATPase and *AE1*. Mutations in these transporters lead to localized supersaturation and crystal deposition, primarily within the medullary interstitium and tubular lumen.

The segmental distribution of the major transport pathways and their associated genetic defects is illustrated in Figure 2, highlighting how abnormalities in distal tubular function give rise to distinct biochemical signatures and characteristic patterns of nephrocalcinosis.

### 4.4. Disorders of Oxalate Metabolism

Primary hyperoxaluria represents the prototypical metabolic cause of monogenic nephrocalcinosis, distinguished by systemic overproduction of oxalate that exceeds the kidney’s excretory and buffering capacity. Oxalate is a highly insoluble anion that avidly complexes with calcium, and even modest increases in urinary oxalate markedly reduce calcium solubility, thereby driving calcium oxalate supersaturation [19,35,83]. In primary hyperoxaluria, this process is amplified to an extreme degree: hepatic defects in glyoxylate metabolism lead to continuous endogenous oxalate generation, resulting in massive filtered loads, progressive tubular deposition, and ultimately widespread medullary and cortical calcium oxalate accumulation [5].

#### 4.4.1. *AGXT*: Primary Hyperoxaluria Type 1 (PH1)

Pathogenic variants in *AGXT*, encoding alanine–glyoxylate aminotransferase, cause the most severe and prevalent form of primary hyperoxaluria. Loss of peroxisomal glyoxylate aminotransferase (AGT) activity diverts glyoxylate metabolism toward oxalate production, leading to sustained systemic oxalosis overproduction and, with declining renal function, to systemic oxalosis [74]. Clinically, patients often present in infancy or childhood with nephrocalcinosis, recurrent nephrolithiasis, progressive kidney failure, and extra-renal oxalate deposition in bone, retina, myocardium, and vasculature [32,49]. The relentless oxalate burden overwhelms tubular transport capacity, induces oxidative stress, and promotes tubular epithelial injury, thereby accelerating crystal adhesion and intrarenal retention (RHPR, Primary Hyperoxaluria Type 2) [77].

#### 4.4.2. *GRHPR*: Primary Hyperoxaluria Type 2 (PH2)

Mutations in *GRHPR*, encoding glyoxylate reductase/hydroxypyruvate reductase, impair cytosolic glyoxylate reduction and lead to elevated urinary excretion of oxalate and L-glycerate. Although typically milder than PH1, primary hyperoxaluria type 2 is frequently associated with recurrent calcium oxalate nephrolithiasis and medullary nephrocalcinosis [73]. Tubular injury arises from the combined effects of oxalate toxicity and intracellular metabolic stress, contributing to progressive crystal deposition and renal impairment [13].

#### 4.4.3. *HOGA1*: Primary Hyperoxaluria Type 3 (PH3)

Defects in *HOGA1*, encoding 4-hydroxy-2-oxoglutarate aldolase, disrupt hydroxyproline metabolism and increase glyoxylate flux toward oxalate production. Primary hyperoxaluria type 3 often presents with recurrent nephrolithiasis in childhood, and nephrocalcinosis may develop when oxalate excretion remains persistently elevated [18]. Although progression to kidney failure is less common than in PH1, the underlying mechanism, namely excessive oxalate delivery to the distal nephron, remains fundamentally the same [19].

However, across all three genetic forms, the unifying pathophysiology is evident: oxalate-driven supersaturation promotes calcium oxalate nucleation, aggregation, and retention within the renal medulla [32,48,83]. Tubular epithelial injury, depletion of endogenous crystallization inhibitors, and impaired washout (particularly within the vulnerable medullary microenvironment described in Section 3) further amplify intrarenal deposition [2,8]. As a result, primary hyperoxaluria represents one of the most potent monogenic drivers of nephrocalcinosis, frequently leading to early and progressive renal damage if not promptly recognized and treated [13,49,50].

### 4.5. Mitochondrial and Metabolic Disorders

Defects in mitochondrial energy production and intermediary metabolism constitute an increasingly recognized group of monogenic contributors to nephrocalcinosis. The kidney is one of the most mitochondria-rich organs, and tubular transport, particularly in the proximal tubule and thick ascending limb, relies heavily on oxidative phosphorylation to sustain ATP-dependent pumps and exchangers [19]. When mitochondrial function is compromised, ATP availability declines, leading to impaired activity of key transporters involved in calcium, phosphate, and proton handling [84]. In parallel, dysfunctional respiratory chain activity increases the generation of reactive oxygen species, promoting oxidative damage to lipids, proteins, and mitochondrial DNA [85]. This combination of bioenergetic failure and oxidative stress renders tubular epithelial cells highly susceptible to injury, loss of polarity, and detachment, thereby facilitating crystal adhesion and retention [77].

Pathogenic variants affecting nuclear transcriptional regulators of metabolic pathways provide a mechanistic link between altered cellular energetics and tubular dysfunction. Mutations in *HNF4A*, a transcription factor controlling networks of genes involved in glucose and lipid metabolism as well as proximal tubular function, can secondarily disturb mitochondrial homeostasis and solute transport [74,86]. These defects impair epithelial energy balance and transporter expression, predisposing to tubular stress and mineral supersaturation.

Similarly, pathogenic variants in mitochondrial DNA, including mitochondrial tRNA genes and other components of the oxidative phosphorylation (OXPHOS) machinery, directly impair mitochondrial protein synthesis and electron transport chain assembly, resulting in reduced ATP generation [87]. Clinically, such disorders often present with a combination of tubulointerstitial nephropathy, incomplete or overt tubular dysfunction (including Fanconi-like features), and radiologic evidence of nephrocalcinosis [5,37]. The renal phenotype reflects the high energetic demands of tubular epithelial cells and their vulnerability to mitochondrial dysfunction.

In this context, nephrocalcinosis is not merely a consequence of isolated electrolyte imbalance but reflects a manifestation of broader bioenergetic failure of the tubular epithelium. Impaired active transport, oxidative injury, and reduced regenerative capacity converge to create a microenvironment that favors calcium salt supersaturation, crystal adhesion, and progressive intrarenal deposition [7,19]. Mitochondrial and metabolic disorders therefore occupy a distinct position within the genetic landscape of nephrocalcinosis, linking cellular energy metabolism directly to crystal-driven kidney injury.

### 4.6. Ciliopathies and Developmental Disorders

Genes that regulate ciliary structure, signaling pathways, and nephron developmental programs play a central role in maintaining tubular architecture, lumen diameter, and coordinated solute transport. When these genes are disrupted, the resulting defects extend far beyond gross structural abnormalities: they alter mechanosensation, epithelial polarity, and flow-dependent signaling, thereby creating a microenvironment that strongly favors crystal retention and nephrocalcinosis [19,88].

Primary cilia function as mechanosensory organelles that detect luminal flow and translate mechanical stimuli into intracellular signaling pathways governing epithelial differentiation, planar cell polarity, and transport regulation. Mutations in *NPHP* genes (nephronophthisis spectrum) or *BBS* genes (Bardet–Biedl syndrome) impair ciliary assembly or intraflagellar transport, leading to shortened, absent, or dysfunctional cilia [89]. These abnormalities compromise flow sensing and disrupt the alignment and orientation of tubular epithelial cells, producing irregular luminal contours and regions of low shear stress [57]. Such microanatomic conditions act as niduses for crystal adhesion, particularly within the medullary collecting ducts, where tubular flow is physiologically slow and crystal washout is inherently limited [77].

Transcriptional regulators such as *HNF1B* further integrate developmental and ciliary pathways. *HNF1B* controls the expression of genes involved in nephron segmentation, tubular morphogenesis, and ion transport [86]. Loss-of-function variants lead to abnormalities in tubular diameter, cyst formation, and impaired reabsorption of magnesium, bicarbonate, and other solutes [5]. These defects alter tubular fluid composition and create zones of stasis that facilitate the persistence, aggregation, and intrarenal retention of calcium phosphate or calcium oxalate crystals [90]. In many affected individuals, medullary nephrocalcinosis represents one of the earliest radiologic findings, often preceding overt declines in renal function [45].

Across ciliopathies and developmental disorders, a unifying pathogenic theme emerges: disruption of the finely tuned relationship between tubular structure, flow dynamics, and solute handling. When this balance is lost, even mild metabolic or electrolyte disturbances can translate into disproportionate crystal retention [57,58,91]. Consequently, ciliary and developmental genes constitute a major category within the monogenic landscape of nephrocalcinosis, linking defects in renal morphogenesis and mechanosensation directly to crystal-driven kidney injury [10,39,92].

### 4.7. Structural and Cystic Kidney Disorders

Abnormalities in tubular morphology and cyst formation represent an important structural pathway through which monogenic disorders predispose to nephrocalcinosis. When the normal architecture of the nephron is distorted, the finely regulated relationships among tubular diameter, flow velocity, and solute transport are disrupted. These alterations generate regions of low shear stress and luminal stasis, conditions that strongly favor the adhesion, persistence, and intrarenal accumulation of calcium phosphate or calcium oxalate crystals, particularly within the medulla [19].

Mutations in *PKHD1*, the gene responsible for autosomal recessive polycystic kidney disease (ARPKD), exemplify this mechanism. *PKHD1* encodes fibrocystin, a ciliary-associated protein essential for collecting duct morphogenesis and planar cell polarity [92]. Loss of fibrocystin leads to fusiform dilatation of the collecting ducts, expansion of the medullary interstitium, and progressive cyst formation [93]. These structural abnormalities slow tubular flow and impair the clearance of crystals once nucleation has occurred. In addition, altered ductal geometry increases the epithelial surface area available for crystal attachment, while interstitial fibrosis compromises medullary perfusion and oxygenation, further amplifying the lithogenic microenvironment [33,77].

Although less common in childhood, mutations in *PKD1* and *PKD2* associated with autosomal dominant polycystic kidney disease (ADPKD) can produce similar effects. Progressive cyst expansion compresses adjacent nephrons, distorts tubular continuity, and disrupts normal solute transport [5,94]. Importantly, even before a substantial cyst burden develops, early defects in ciliary signaling and epithelial polarity may induce subtle alterations in flow dynamics that promote crystal retention [95]. As cysts enlarge, medullary stasis becomes more pronounced, raising the likelihood of nephrocalcinosis or recurrent nephrolithiasis.

Across structural and cystic kidney disorders, a unifying theme emerges: architectural distortion of the nephron (whether through cyst formation, tubular dilation, or loss of epithelial polarity) creates a physical environment in which crystals can form, adhere, and accumulate [19,45]. This structural pathway acts synergistically with metabolic and transport-related mechanisms, underscoring the multifactorial nature of nephrocalcinosis in monogenic kidney disease.

Taken together, the monogenic disorders outlined in this chapter illustrate how diverse molecular lesions (affecting ion transport, mineral metabolism, oxalate handling, mitochondrial energetics, ciliary signaling, and nephron architecture) converge on a limited set of pathophysiological pathways culminating in nephrocalcinosis [1,2,33]. Despite increasingly well-defined genotype–phenotype correlations, substantial overlap in biochemical profiles and clinical presentations often limits the ability to infer the underlying genetic defect based solely on clinical and laboratory evaluation [4,11]. This diagnostic ambiguity highlights the importance of systematic and timely genomic testing, particularly in pediatric and consanguineous populations, where monogenic etiologies are enriched [3,7]. A structured overview of the principal genes, their molecular functions, and their characteristic biochemical signatures is summarized in Table 2, highlighting the extent of this heterogeneity and reinforcing the necessity of a genetics-guided diagnostic approach.

As the spectrum of implicated genes continues to expand, the selection of the appropriate genetic testing modality becomes a critical step in the clinical work-up of nephrocalcinosis. Targeted gene panels, whole-exome sequencing, and whole-genome sequencing have markedly increased diagnostic yield and revealed unexpected molecular diagnoses with direct implications for management [1,5]. These considerations provide the rationale for transitioning from a mechanistic understanding to a structured discussion of contemporary genetic testing strategies, which now form the backbone of precision nephrology in nephrocalcinosis [18,45].

## 5. Genotype–Phenotype Correlations in Monogenic Nephrolithiasis

Genotype–phenotype correlations in monogenic nephrolithiasis arise from the convergence of diverse defects onto a limited number of biochemical signatures and stone-forming microenvironments [5,10,44]. Although the underlying genetic lesions affect distinct pathways, including electrolyte transport, acid–base regulation, oxalate metabolism, mitochondrial energetics, and ciliary signaling, the resulting clinical and laboratory phenotypes cluster into recognizable patterns [1,11,33]. The mechanistic categories, summarized in Table 3, provide a structural framework for linking clinical presentation, biochemical abnormalities, and extrarenal manifestations to specific molecular defects. A comprehensive gene list with OMIM IDs, inheritance, clinical features and concise mechanisms is provided in Table 4.

Monogenic nephrocalcinosis is therefore characterized by recurring clinical and biochemical “signatures” that reflect the dominant pathogenic mechanism. Recognition of these patterns allows clinicians to infer likely genetic etiologies early in the diagnostic process, an approach that is particularly valuable in pediatric patients, where timely molecular confirmation can substantially influence management and long-term outcomes [9].

### 5.1. Age at Onset as a Diagnostic Clue

The age at which nephrocalcinosis first becomes apparent provides one of the most informative indicators of the underlying pathogenic pathway.

Neonatal-onset nephrocalcinosis is typically associated with disorders causing severe and immediate disturbances in tubular ion handling or oxalate metabolism. Distal renal tubular acidosis due to *ATP6V1B1* or *ATP6V0A4* mutations often manifests within the first months of life with failure to thrive, vomiting, and profound metabolic acidosis, and early medullary nephrocalcinosis [111]. Familial hypomagnesemia with hypercalciuria and nephrocalcinosis (*CLDN16*, *CLDN19*) similarly presents in infancy, reflecting marked magnesium wasting and hypercalciuria [16]. In extreme cases, primary hyperoxaluria type 1 (*AGXT*) may present neonatally with nephrocalcinosis and nephromegaly when oxalate overproduction is severe [112].

Early childhood-onset nephrocalcinosis more commonly reflects developmental or ciliary defects. *HNF1B*-related disease typically presents during early childhood with renal cysts, hypomagnesemia, and medullary nephrocalcinosis [113]. *NPHP1*-related nephronophthisis manifests with polyuria, salt wasting, and progressive medullary echogenicity, often before overt renal insufficiency [114].

Adolescent or adult-onset nephrocalcinosis is more characteristic of milder transport defects or heterozygous states. Individuals carrying monoallelic *SLC34A1* or *SLC34A3* variants may present later with hypercalciuria, nephrolithiasis, or subtle nephrocalcinosis. Likewise, mild activating or inactivating *CASR* variants may remain clinically silent until adolescence or adulthood, when disturbances in calcium homeostasis become evident [115].

### 5.2. Biochemical Signatures

Distinct biochemical constellations serve as a mechanistic fingerprint that frequently narrows the differential diagnosis to a limited set of genes.

Hypercalciuria-dominant phenotypes arise from defects that increase distal calcium delivery or impair calcium reabsorption. Mutations in *CLDN16* and *CLDN19* disrupt paracellular divalent cation transport in the thick ascending limb, producing severe hypercalciuria and early medullary nephrocalcinosis [116]. Activating *CASR* variants suppress both *TAL* paracellular calcium transport and distal *TRPV5*-mediated reabsorption, resulting in hypocalcemia with marked renal calcium wasting [117]. Defects in *SLC34A1* and *SLC34A3* increase 1,25-dihydroxyvitamin D synthesis, enhancing intestinal calcium absorption and filtered calcium load, thereby promoting hypercalciuria despite systemic hypophosphatemia [118].

Hypomagnesemia-dominant phenotypes point toward defects in magnesium handling within the *TAL* or distal convoluted tubule. Profound hypomagnesemia is characteristic of FHHNC caused by *CLDN16/19* mutations. In *HNF1B*-related disease, hypomagnesemia results from the transcriptional downregulation of *FXYD2*, leading to impaired Na^+^/K^+^-ATPase activity and reduced magnesium uptake in the distal nephron [117].

Hyperoxaluria-dominant phenotypes are pathognomonic for primary hyperoxaluria types 1–3. Mutations in *AGXT*, *GRHPR*, and *HOGA1* lead to systemic oxalate overproduction that overwhelms renal excretory capacity and drives calcium oxalate deposition [50]. Urinary oxalate levels are markedly elevated and often accompanied by nephromegaly and progressive nephrocalcinosis.

Alkaline urine phenotypes strongly suggest impaired distal acidification. Distal renal tubular acidosis caused by *ATP6V1B1* or *ATP6V0A4* mutations produces persistently alkaline urine (pH > 6.5), hypocitraturia, and calcium phosphate supersaturation [14]. Certain ciliopathies, such as *OFD1*-related disease, may present with an incomplete dRTA phenotype and similar urinary alkalinity.

Polyuria-driven concentration defects are characteristic of ciliopathies, including *NPHP1*, intraflagellar transport (*IFT*) gene defects, and ADPKD. Although increased urine flow might be expected to enhance crystal clearance, recurrent dehydration episodes paradoxically concentrate calcium and oxalate and reduce the effectiveness of crystallization inhibitors, thereby promoting nephrocalcinosis [58].

### 5.3. Extrarenal Features as Diagnostic Anchors

Extrarenal manifestations often provide decisive clues to the underlying genetic etiology, particularly when renal findings are nonspecific.

Ocular involvement is a hallmark of *CLDN19*-related FHHNC, where macular coloboma, nystagmus, or visual impairment accompany the renal phenotype [119]. This distinguishes *CLDN19* from *CLDN16*, which lacks ocular features [70].

Skeletal manifestations are characteristic of *SLC34A3*-related hereditary hypophosphatemic rickets with hypercalciuria (HHRH). Defective phosphate reabsorption leads to rickets, bone pain, and pathological fractures [79]. In addition, ciliopathies such as short-rib thoracic dysplasia (linked to *IFT88* and *IFT140* mutations) present with a distinct skeletal dysplasia, including thoracic narrowing and limb shortening, reflecting the fundamental role of cilia in chondrocyte signaling [10,39,92].

Pancreatic and metabolic features are strongly associated with *HNF1B* mutations. Affected individuals may exhibit pancreatic hypoplasia, early-onset diabetes, elevated liver enzymes, or genital tract malformations alongside renal cysts and nephrocalcinosis [120].

## 6. Diagnostic Approach

A structured diagnostic strategy is essential for distinguishing monogenic nephrocalcinosis from multifactorial causes and for directing appropriate genetic testing [18,45]. Because many monogenic disorders present early in life and share overlapping imaging features, diagnosis cannot rely on radiologic appearance alone. Instead, an integrated, mechanism-oriented approach is required, combining age at onset, biochemical signatures, imaging patterns, and syndromic features to guide rational genetic evaluation [3,5].

### 6.1. Clinical Assessment

The diagnostic process begins with clinical stratification aimed at narrowing the underlying pathogenic category. Age at onset provides an early anchor: neonatal presentations suggest severe defects in acid–base regulation, magnesium handling, or oxalate metabolism [6,50], whereas early childhood onset more commonly points toward developmental or ciliary disorders [10,92]. Later onset raises the possibility of milder transport defects or heterozygous states [17,63].

A detailed family history, including consanguinity and early-onset kidney disease, or unexplained renal failure, increases the likelihood of recessive monogenic etiologies [4,11]. Associated symptoms such as polyuria, growth delay, recurrent vomiting, nephrolithiasis, or bone pain further refine the differential diagnosis by implicating specific tubular or metabolic pathways [1,33].

### 6.2. Biochemical Evaluation

Biochemical profiling represents the most powerful discriminator in monogenic nephrocalcinosis. Serum and urine profiles often reveal characteristic constellations that map directly onto underlying molecular defects [2,3].

Hypercalciuria accompanied by hypomagnesemia suggests *CLDN16/CLDN19*-related FHHNC [16,116], whereas hypercalciuria with hypophosphatemia and elevated 1,25-dihydroxyvitamin D points toward *SLC34A1* or *SLC34A3* defects [54,73]. Marked hyperoxaluria is diagnostic for primary hyperoxaluria, while persistently alkaline urine with hypocitraturia indicates distal renal tubular acidosis. In contrast, polyuria with low urine osmolality suggests a concentrating defect typical of ciliopathies such as *NPHP1*-related disease [120]. These biochemical signatures often allow clinicians to prioritize a focused set of candidate genes before genetic testing is initiated, improving diagnostic efficiency and yield.

### 6.3. Imaging Patterns

Renal ultrasound remains the first-line imaging modality for nephrocalcinosis and provides additional diagnostic clues when interpreted in the clinical context [4,82]. Medullary nephrocalcinosis is the predominant pattern across most monogenic disorders, reflecting the intrinsic vulnerability of the medulla.

Specific imaging combinations can be informative. Medullary echogenicity associated with renal cysts suggests *HNF1B*-related disease [113,121], whereas medullary nephrocalcinosis accompanied by nephromegaly raises suspicion for primary hyperoxaluria [112]. Cortical nephrocalcinosis is less common but may be observed in severe hyperoxaluria or advanced metabolic disease. In ciliopathies, nephrocalcinosis frequently coexists with increased medullary echogenicity and progressive corticomedullary differentiation loss [88,89].

### 6.4. Genetic Testing Strategy

Genetic testing is central to diagnostic confirmation and subsequent management. The choice of testing modality depends on the clarity of the biochemical and clinical phenotype. When biochemical signatures strongly implicate a specific pathway, for example, hyperoxaluria, distal renal tubular acidosis, or FHHNC, targeted gene panels provide rapid and cost-effective diagnosis.

In patients with atypical presentations, syndromic features, or negative panel results, whole-exome sequencing (WES) or whole-genome sequencing (WGS) substantially increase diagnostic yield and may reveal unexpected or novel molecular diagnoses [5]. Interpretation of genetic variants requires careful integration with biochemical and clinical data, as incomplete penetrance and variable expressivity are common across snephrocalcinosis-associated genes [7].

### 6.5. Mechanism-Based Diagnostic Framework

A mechanism-based diagnostic framework integrates clinical presentation, biochemical findings, and imaging patterns to guide genetic testing [19]. Early-onset disease with severe electrolyte disturbances directs attention toward primary transport disorders [16]; hyperoxaluria mandates evaluation for primary hyperoxaluria; alkaline urine with growth failure suggests distal renal tubular acidosis [14]; and polyuria with concentrating defects points toward ciliopathies. This structured approach reduces diagnostic delay, improves the accuracy of genetic testing, and facilitates the timely initiation of mechanism-directed therapies [45,60].

The transition from clinical suspicion to genetic confirmation relies on identifying a dominant biochemical signature, which serves as the primary branching point for differential diagnosis (Figure 1) [3,4]. Within this framework, the diagnostic pathway typically progresses through four principal analytical branches:

*(i)*  *Electrolyte-Driven Branch.* Early-onset presentations characterized by severe electrolyte disturbances (e.g., hypokalemia, metabolic alkalosis) suggest primary transport disorders. Priority genes include *SLC12A1*, *KCNJ1*, *CLCNKB*, *BSND*, and *SLC12A3*, best assessed using targeted next-generation sequencing panels or functional assays such as patch-clamp analysis [17,33];
*(ii)*  *Oxalate Metabolism Branch.* Elevated urine oxalate levels are pathognomonic for primary hyperoxaluria, and prompt testing of *AGXT* (PH1), *GRHPR* (PH2), and *HOGA1* (PH3). Confirmatory investigations may include plasma oxalate or glycolate measurement, and, in selected cases, liver-based enzymatic assessment [13,49,56,122];
*(iii)*  *Acid-Base Branch.* Persistent alkaline urine (pH > 5.5) associated with growth failure is a characteristic of distal renal tubular acidosis. Genetic prioritization focuses on *SLC4A1*, *ATP6V1B1*, and *ATP6V0A4,* often supported by urine acidification tests such as the furosemide–fludrocortisone challenge [14,15,51,52].
*(iv)*  *Ciliary and Developmental Branch*. Childhood-onset polyuria with concentrating defects suggests a ciliopathy involving cilia structure or signaling. Genetic testing should encompass the *NPHP* gene family, *NEK8*, *TMEM67*, and *WDR19*. Diagnostic confirmation relies on associated extrarenal features and, in selected cases, renal biopsy with ultrastructural analysis [6,10,39].

**Figure 3:**
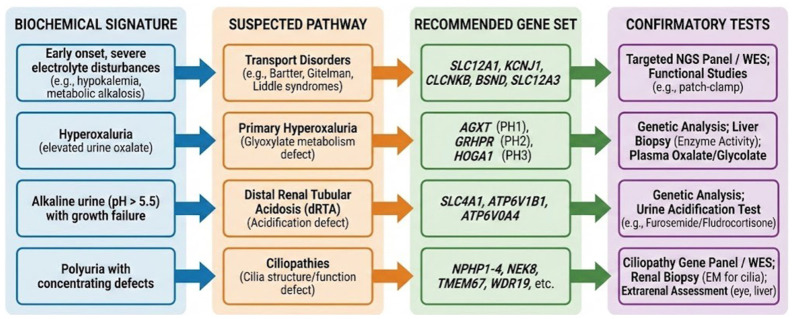
Integrated Diagnostic Algorithm for Monogenic Nephrocalcinosis. A hierarchical flowchart mapping initial biochemical signatures to suspected molecular pathways, recommended gene sets for targeted analysis, and definitive confirmatory tests. This approach enables a precision medicine strategy by correlating metabolic fingerprints with specific genetic etiologies.

By applying this hierarchical framework, clinicians can efficiently navigate the genetic complexity of monogenic nephrocalcinosis (Figure 3). This structured approach ensures that high-yield genetic testing is performed early, allowing for personalized management strategies that address the specific underlying molecular defect [5,18].

## 7. Therapeutic Implications and Precision Medicine Approaches

Therapeutic decision-making in monogenic nephrocalcinosis increasingly depends on understanding the molecular mechanism driving crystal formation. While supportive measures such as hydration and citrate supplementation remain foundational, genotype and pathway-directed therapies have transformed outcomes for several inherited disorders [1,61]. The overarching therapeutic goal is to interrupt the specific biochemical cascade that leads to tubular supersaturation, epithelial injury, and crystal retention, thereby slowing or preventing progression to chronic kidney disease [2,31].

### 7.1. Mechanism-Directed Therapies

#### 7.1.1. Oxalate Metabolism Disorders

Disorders of oxalate metabolism represent the clearest, most advanced paradigm of precision therapy in nephrocalcinosis. In primary hyperoxaluria type 1, RNA interference therapy targeting hepatic glycolate oxidase markedly reduces endogenous oxalate production and lowers the filtered oxalate load [12,49]. Pyridoxine supplementation remains effective in a subset of patients with *AGXT* variants that preserve cofactor responsiveness [122]. In advanced disease stages, combined liver–kidney transplantation remains the definitive intervention, as hepatic correction is required to normalize oxalate metabolism and prevent systemic oxalosis [48,50].

#### 7.1.2. Distal Acidification Defects and Alkali Therapy

Monogenic forms of distal renal tubular acidosis respond to chronic alkali therapy, which corrects systemic acidosis, reduces urinary calcium excretion, and increases citrate availability [59]. Potassium citrate is preferred, as it simultaneously addresses hypocitraturia and hypokalemia. Early and sustained correction of acidosis improves growth outcomes and significantly reduces progression of nephrocalcinosis, particularly in pediatric patients [15].

#### 7.1.3. Calcium and Magnesium Transport Disorders

Disorders affecting divalent cation handling require targeted supplementation strategies. In familial hypomagnesemia with hypercalciuria and nephrocalcinosis, sustained magnesium replacement partially restores inhibitory control over calcium crystallization, although renal wasting often limits full biochemical normalization [16,64]. Thiazide diuretics may reduce hypercalciuria in selected cases, but therapeutic response is variable due to the primary paracellular defect. In *HNF1B*-related disease, magnesium supplementation improves biochemical abnormalities and neuromuscular symptoms but does not reverse the underlying transcriptional dysregulation [113,121].

#### 7.1.4. Genetic Defects of Phosphate Transport

Phosphate-wasting disorders benefit from oral phosphate supplementation combined with careful modulation of calcitriol exposure. Restoration of serum phosphate attenuates the compensatory increase in 1,25-dihydroxyvitamin D, thereby reducing intestinal calcium absorption and filtered calcium load [79]. This strategy lowers hypercalciuria and may slow the progression of nephrocalcinosis when initiated early [54].

#### 7.1.5. Calcium-Sensing Receptor Disorders

Calcium-sensing receptor-related disorders require nuanced management. Activating *CASR* variants should not be treated with excessive calcium or vitamin D supplementation, as this exacerbates hypercalciuria and nephrocalcinosis risk [65]. Emerging CaSR antagonists (calcilytics) offer a potential future therapeutic avenue by restoring more physiological tubular calcium handling [117].

#### 7.1.6. Ciliary Dysfunction, Tubular Flow and Supportive Care

Ciliopathies currently lack pathway-specific pharmacologic therapies. Management, therefore, focuses on mitigating downstream consequences of tubular flow defects and concentrating abnormalities. High fluid intake, citrate supplementation, and avoidance of dehydration reduce crystal retention and secondary injury [57]. Early identification of progressive renal dysfunction allows timely nephrology follow-up and preparation for renal replacement therapy when necessary [92,115].

### 7.2. General Principles of Crystal Prevention

Across all monogenic etiologies, several universal interventions reduce the physicochemical drivers of nephrocalcinosis. Adequate hydration lowers tubular solute concentration and reduces supersaturation [34]. Citrate supplementation inhibits nucleation and aggregation of both calcium phosphate and calcium oxalate crystals while improving urinary buffering capacity [30]. Dietary counseling addresses oxalate intake, sodium load, and calcium balance [32]. Avoidance of nephrotoxins and careful monitoring of medications that alter urinary pH or solute excretion are particularly important in genetically susceptible patients [2].

### 7.3. Precision Nephrology in Clinical Practice

Molecular diagnosis increasingly shapes long-term management in nephrocalcinosis. Identification of the causal gene informs prognosis, as some disorders progress rapidly to chronic kidney disease while others remain stable for decades [10,44]. Genetic confirmation guides family counseling, reproductive planning, and cascade testing [11]. In transplant planning, specific diagnoses, such as primary hyperoxaluria or distal renal tubular acidosis, directly influence organ selection and peri-transplant management strategies. Enrollment in clinical trials for emerging therapies is increasingly genotype-dependent, particularly for RNA-based or gene-targeted interventions [12,13].

Precision nephrology thus reframes nephrocalcinosis from a nonspecific radiologic finding to a molecularly defined disease spectrum with actionable therapeutic pathways. Early genetic testing enables the timely initiation of mechanism-directed therapy, improves outcomes, and reduces the burden of irreversible renal damage [18,45].

## 8. Integrative Perspectives and Future Directions in Precision Nephrology

Advances in molecular nephrology have reframed nephrocalcinosis from a nonspecific radiologic finding into a heterogeneous phenotype arising from discrete genetic, metabolic, and microenvironmental disturbances [1,2,33]. Across disorders of tubular transport, mineral metabolism, oxalate handling, mitochondrial energetics, and ciliary signaling, a unifying principle has emerged: diverse molecular lesions converge on a limited set of physicochemical and biological pathways that promote supersaturation, crystal nucleation, epithelial injury, and interstitial remodeling [8,31]. This convergence explains why nephrocalcinosis appears radiologically similar across conditions with markedly different biochemical signatures and clinical trajectories [6,10].

The renal medulla represents a critical amplifier of genetic vulnerability. Hyperosmolality, hypoxia, and low tubular flow create a permissive microenvironment in which even modest metabolic disturbances can trigger crystal formation and retention [2,8]. Developmental and ciliary disorders further distort tubular architecture and impair flow sensing, reinforcing the transition from crystalluria to fixed intrarenal deposits [57,91]. Once crystals adhere to the tubular epithelium, inflammatory and fibrotic pathways accelerate nephron loss, linking nephrocalcinosis directly to long-term renal impairment [31,32].

Despite the expanding catalog of implicated genes, substantial genotype–phenotype variability persists. Penetrance and expressivity are influenced by age, environmental factors, and modifier alleles, complicating clinical interpretation [5,11]. These observations underscore the limitations of imaging-based classification and highlight the need for mechanism-based frameworks integrating genetics, biochemistry, and renal physiology.

### 8.1. Expanding the Genetic Architecture: Beyond Single-Gene Models

Although more than 30 genes are now linked to nephrocalcinosis [10], many patients remain genetically unexplained. Increasing evidence suggests contributions from oligogenic inheritance, modifier alleles, and polygenic risk [3,5]. Future studies using whole-genome sequencing, structural variant detection, and long-read technologies will help uncover noncoding regulatory variants, complex rearrangements, and gene–gene interactions that influence susceptibility [1,4]. Understanding these multilayered contributions will refine variant interpretation and reduce the burden of variants of uncertain significance.

### 8.2. Single-Cell and Spatial Profiling of Tubular Vulnerability

The renal medulla’s unique physiology makes it disproportionately susceptible to crystal injury, yet the cellular determinants of this vulnerability remain incompletely defined. Single-cell RNA sequencing and spatial transcriptomics are beginning to map transporter expression, metabolic states, and stress responses along the nephron with unprecedented resolution [38,39]. These approaches will clarify how specific tubular segments respond to hypercalciuria, hyperoxaluria, or alkaline pH, and may identify novel protective pathways or biomarkers of early injury [30,32].

### 8.3. Organoids and Microfluidic Models for Crystal Kinetics

Traditional in vitro systems cannot replicate the steep osmotic gradients, low oxygen tension, and shear-dependent flow that characterize the medullary microenvironment. Kidney organoids and microfluidic “kidney-on-chip” platforms now allow controlled modeling of crystal nucleation, aggregation, and epithelial adhesion under physiologic flow [34,43]. These systems will enable the mechanistic dissection of how ciliary dysfunction, mitochondrial stress, or transporter defects alter crystal dynamics [37,39] and they provide a platform for testing targeted therapies.

### 8.4. AI-Enhanced Imaging and Phenotype Prediction

Machine learning tools are increasingly capable of detecting subtle medullary echogenicity, quantifying nephrocalcinosis burden, and identifying patterns suggestive of specific genetic etiologies [9,57]. Integrating imaging with biochemical and genomic data may enable automated phenotype clustering and early prediction of monogenic disease [18,45]. Such tools could support earlier referrals for genetic testing and more precise monitoring of disease progression.

### 8.5. Emerging Therapeutics Targeting Molecular Pathways

Precision therapies are expanding rapidly. RNA-based treatments for primary hyperoxaluria have demonstrated the feasibility of pathway-specific intervention [12,13], and similar approaches may emerge for phosphate-wasting disorders or *CaSR*-related disease [65,117]. Mitochondrial protective agents, modulators of crystallization inhibitors, and anti-inflammatory therapies targeting *NLRP3* activation represent additional avenues [8,31]. Gene editing technologies may eventually offer curative strategies for selected recessive disorders, though safety and delivery challenges remain substantial [5].

### 8.6. Longitudinal Registries and Global Collaboration

Given the rarity and heterogeneity of monogenic nephrocalcinosis, large-scale registries and international consortia are essential for defining natural history, treatment response, and genotype–phenotype variability [60,111]. Harmonized data collection, including imaging, biochemistry, genomics, and patient-reported outcomes, will support evidence-based guidelines and accelerate therapeutic development [1,18].

### 8.7. Integrating Precision Nephrology into Routine Care

The future of nephrocalcinosis management lies in embedding genetic evaluation into early clinical workflows, particularly in pediatric populations [3,7]. As sequencing becomes more accessible, clinicians will increasingly rely on molecular diagnosis to guide surveillance, therapy, and family counseling [5,10]. The challenge ahead is to ensure equitable access to testing and to develop clinical decision tools that translate complex genomic data into actionable care pathways [18,45].

## 9. Conclusions

Nephrocalcinosis represents a unifying radiologic manifestation of diverse monogenic and metabolic disorders, yet its underlying biology is profoundly heterogeneous. The mechanisms reviewed herein—tubular supersaturation, epithelial injury, medullary microenvironmental stress, mitochondrial dysfunction, and ciliary signaling defects—demonstrate how distinct molecular lesions converge toward a shared endpoint of intrarenal calcium salt deposition. Recognizing these convergent pathways reframes nephrocalcinosis not as a static imaging finding, but as a dynamic, genetically modulated process shaped by renal physiology and environmental modifiers.

Genotype–phenotype correlations provide a powerful framework for early recognition and rational diagnostic prioritization. Age at onset, biochemical signatures, and extrarenal features frequently point toward specific pathogenic pathways before genetic results become available. Integrating these clinical clues into structured diagnostic algorithms improves the precision of genetic testing, reduces diagnostic delay, and enables timely initiation of mechanism-directed therapies.

The emergence of RNA-based treatments, targeted metabolic interventions, and pathway-specific management strategies illustrates the transformative potential of precision nephrology in altering disease trajectories. Future progress will depend on expanding the genetic architecture of nephrocalcinosis, refining mechanistic models through single-cell and spatial technologies, and developing organoid and microfluidic systems that faithfully replicate the medullary microenvironment. Advances in AI-assisted imaging, combined with global registries and collaborative networks, will further enhance diagnosis, monitoring, and therapeutic development.

Ultimately, the integration of molecular diagnostics with biochemical and clinical phenotyping offers a path toward individualized care, improved prognostication, and better long-term renal outcomes. As sequencing becomes increasingly accessible and targeted therapies continue to evolve, nephrocalcinosis will transition from a descriptive radiologic term into a gateway for precision medicine across pediatric and adult nephrology.

## Figures and Tables

**Table 1:** Epidemiological, genetic, and clinical characteristics of pediatric nephrocalcinosis derived from recent cohort studies and registry data. Values are reported as percentages (%; *n*/*N*) or population estimates, where available, and are stratified according to clinical subgroups.

Category	Key Parameter	Reported Values/Findings	References
General Pediatric Population	Prevalence/Incidence	Precise population-wide rates are lacking; increasing incidence of nephrolithiasis; NC prevalence is likely underreported in registries.	[21,24]
Preterm Infants	Ultrasound Screening Incidence	7–41% (variable across studies); typical incidence in very-preterm cohorts: ~15%; VLBW cohorts: up to 35–40%.	[22,25]
	Natural History	Frequent spontaneous resolution, typically by 6–8 months of age.	[22,25]
Etiological Workup	Diagnostic Yield (Standard Metabolic)	>75% of pediatric cases have an identifiable clinical or biochemical cause after standard evaluation.	[21,26]
Genetic Diagnostics	Targeted Panel (Multicenter)	32% overall yield; 40% yield in the nephrocalcinosis subgroup (e.g., Invitae panel, *n* = 113).	[21,24]
	WES/WGS (Selected Cohorts)	20–70% yield depending on selection; up to ~70% in specific tubulopathies; 20–40% for general NC/NL cohorts.	[21,24]
Primary Hyperoxaluria (PH)	Genetic Prevalence (gnomAD)	PH1 (*AGXT*): ~1:209,357; PH2 (*GRHPR*): ~1:863,028; PH3 (*HOGA1*): ~1:90,834.	[21,27]
	Subtype Distribution	PH1 accounts for 70–85% of genetically diagnosed cases.	[21,27]
	Clinical Outcome (PH1)	High risk of ESKD; historically >70% progression without targeted therapy (improving with RNAi therapies).	[21,27]
FHHNC	Estimated Prevalence	Ultra-rare: <1/1,000,000 (Orphanet); ~200 cases described in the literature.	[21,22]
	Genetic and Clinical Features	*CLDN16* and *CLDN19* mutations; *CLDN19* often involves ocular defects; ~50% progress to RRT by the second decade.	[21,22]
Distal RTA (dRTA)	Genetic Basis	Mutations in *ATP6V1B1*, *ATP6V0A4*, or *SLC4A1*.	[21,27]
	Prognosis	Generally good with alkali therapy; late diagnosis or specific variants increase the risk of CKD.	[21,27]
Other Common Genes	High-Frequency Findings	*SLC34A3* (HHRH), *HOGA1*, *SLC34A1*, *CASR*, and *TRPV5* are frequently identified in multigene panels.	[21,24]
Clinical Impact	Management Change	Genetic diagnosis leads to clinical management changes in 20–40% of cases (therapy, transplant planning, counseling).	[21,24]

**Table 2:** Key Genetic Determinants of Nephrocalcinosis: Protein Function and Phenotypic Correlates.

Gene	Protein/Function	Primary Biochemical Phenotype	Key Extra-Renal Features	References
*CASR*	Calcium-sensing receptor	Hypercalcemia, Hypocalciuria (FHH) or Hypocalcemia, Hypercalciuria (ADH)	Possible parathyroid dysfunction	[96]
*CLDN16/CLDN19*	Claudins (Paracellular transport)	Hypomagnesemia, Hypercalciuria (FHHNC)	Severe visual impairment (specifically for *CLDN19*)	[97]
*KCNJ1/SLC12A1*	ROMK/NKCC2 (Henle’s Loop)	Hypokalemic metabolic alkalosis (Bartter Syndrome)	Polyhydramnios, growth retardation	[98,99]
*ATP6V1B1/SLC4A1*	H^+^-ATPase/Anion exchanger 1	Hypercalciuria, distal Renal Tubular Acidosis (dRTA)	Sensorineural hearing loss (*ATP6V1B1*)	[52]
*AGXT*	Alanine-glyoxylate aminotransferase	Severe Hyperoxaluria (PH1)	Systemic oxalosis (bone, heart, skin)	[100,101]
*SLC34A1/SLC34A3*	NaPi-IIa/NaPi-IIc (Phosphate transporters)	Hypophosphatemia, Hypercalciuria	Rickets, Osteomalacia	[102,103]
*CYP24A1*	25-hydroxyvitamin D 24-hydroxylase	Hypercalcemia, suppressed PTH, high 1,25(OH)_2_D	Hypersensitivity to Vitamin D	[104,105]
*OCRL*	Phosphatidylinositol 4,5-bisphosphate 5-phosphatase	Low-molecular-weight proteinuria, Hypercalciuria	Cataracts, intellectual disability (Lowe Syndrome)	[106,107]
*HNF1B*	Transcription factor	Hypomagnesemia, Hyperuricosuria	MODY5 diabetes, genital tract malformations	[108,109]
*PKD1/PKD2*	Polycystin 1 and 2	Electrolyte imbalances (secondary to cyst formation)	Polycystic liver disease, intracranial aneurysms	[110]

**Table 3:** Summary of Genetic Pathways and Mechanisms of Nephrocalcinosis.

Category	Primary Gene(s)	Key Biochemical Signature	Pathophysiological Mechanism
Divalent Cation Transport	*CLDN16*, *CLDN19*, *CASR*	Hypomagnesemia, Hypercalciuria	Loss of paracellular cation reabsorption in TAL; increased distal calcium load.
Phosphate Wasting	*SLC34A1*, *SLC34A3*	Hypophosphatemia, High 1,25(OH)_2_D	Dysregulated Vit D axis increases intestinal Ca absorption and urinary Ca/Pi product.
Acid-Base Regulation	*ATP6V1B1*, *ATP6V0A4*, *SLC4A1*	Persistent Alkaline Urinary pH (pH > 6.0)	Distal RTA; calcium phosphate (apatite) supersaturation in the distal nephron.
Oxalate Metabolism	*AGXT*, *GRHPR*, *HOGA1*	Severe Hyperoxaluria	Systemic oxalate overproduction; calcium oxalate precipitation and tubular injury.
Mitochondrial/Metabolic	*HNF4A*, *mtDNA*, *HOGA1*	Variable; High Oxidative Stress	ATP depletion impairs active transport; ROS-induced epithelial “stickiness.”
Ciliary and Developmental	*HNF1B*, *NPHP* genes, *BBS* genes	Hypomagnesemia (*HNF1B*), Polyuria	Impaired mechanosensing and low-shear environments promote crystal retention.
Structural/Cystic	*PKHD1*, *PKD1*, *PKD2*	Hypocitraturia, Tubular stasis	Architectural distortion and stagnant flow favor crystal nucleation and trapping.

**Table 4:** List of genes implicated in nephrocalcinosis and related renal mineral disorders, with OMIM IDs, inheritance patterns, typical clinical features and brief mechanistic explanations.

Gene	Location	OMIM ID	Disease (OMIM Name)	Inheritance	Short Clinical Features	Concise Molecular Mechanism
*CLDN16*	3q28	603959	Renal hypomagnesemia 3	AR	Hypomagnesemia, hypercalciuria, early nephrocalcinosis, progressive CKD; childhood onset	Loss-of-function in claudin 16 disrupts paracellular Mg^2+^/Ca^2+^ reabsorption in the TAL, increasing distal Ca delivery and promoting crystal formation
*CLDN19*	1p34.2	610036	Renal hypomagnesemia 5	AR	As *CLDN16* plus severe ocular abnormalities (retinal defects); childhood onset	Claudin 19 defect impairs paracellular Mg^2+^/Ca^2+^ transport in TAL and affects retinal development, explaining ocular comorbidity
*SLC12A1*	15q21.1	600839	Bartter syndrome type 1	AR	Antenatal/neonatal polyhydramnios, salt wasting, metabolic alkalosis, hypercalciuria, nephrocalcinosis	NKCC2 (*SLC12A1*) loss reduces TAL salt reabsorption, alters lumen voltage and increases Ca^2+^ delivery distally
*SLC12A3*	16q13	600968	Gitelman syndrome	AR	Hypokalemia, hypomagnesemia, hypocalciuria or variable Ca handling; usually later childhood/adolescence	Thiazide-sensitive NaCl cotransporter defect in DCT; alters electrolyte balance and can secondarily affect Ca/Mg handling
*SLC34A1*	5q35.3	182309	*SLC34A1*-related phosphate wasting	AD	Hypophosphatemia, elevated 1,25(OH)_2_D, hypercalciuria, nephrocalcinosis; infant–childhood	NaPi-IIa proximal tubule transporter defect → renal phosphate wasting → compensatory ↑ 1,25(OH)_2_D and increased intestinal Ca absorption
*SLC34A3*	9q34.3	609826	HHRH (hereditary hypophosphatemic rickets with hypercalciuria)	AR	Hypophosphatemic rickets, hypercalciuria, nephrocalcinosis; childhood	NaPi-IIc defect → renal phosphate loss → ↑ 1,25(OH)_2_D → increased Ca absorption and hypercalciuria
*AGXT*	2q37.3	604285	Primary hyperoxaluria type 1	AR	Marked hyperoxaluria, recurrent stones, early diffuse nephrocalcinosis, progressive CKD; systemic oxalosis in advanced disease	*AGT* deficiency diverts glyoxylate to oxalate → hepatic overproduction of oxalate and massive renal oxalate load
*GRHPR*	9p13.2	604296	Primary hyperoxaluria type 2	AR	Hyperoxaluria, recurrent stones, nephrocalcinosis; variable severity	*GRHPR* deficiency impairs glyoxylate reduction → increased oxalate production
*HOGA1*	10q24.2	613597	Primary hyperoxaluria type 3	AR	Recurrent stones, nephrocalcinosis; often milder course than PH1	*HOGA1* defect perturbs hydroxyproline metabolism, increasing glyoxylate flux toward oxalate
*ATP6V1B1*	2p13.3	192132	Distal RTA with deafness	AR	Distal RTA, early nephrocalcinosis, growth failure, sensorineural hearing loss (in many cases)	V-ATPase B1 subunit defect impairs proton secretion by α-intercalated cells → alkaline urine, hypocitraturia and calcium phosphate precipitation
*ATP6V0A4*	7q34	605239	Distal RTA 3	AR	Distal RTA, nephrocalcinosis; hearing loss variable	V-ATPase A4 subunit defect reduces proton translocation in collecting duct intercalated cells → impaired urinary acidification
*SLC4A1*	17q21.31	109270	*AE1*-related distal RTA	AD/AR	Incomplete/complete dRTA, nephrocalcinosis; variable onset	*AE1* (Cl^−^/HCO_3_^−^ exchanger) defects impair bicarbonate handling in intercalated cells, reducing luminal proton secretion
*CASR*	3q13.33-q21.1	601199	Calcium-sensing receptor disorders	AD	Syndromes range from familial hypocalciuric hypercalcemia to autosomal dominant hypocalcemia; nephrocalcinosis may occur with hypercalciuria	Gain- or loss-of-function alters parathyroid and renal Ca sensing, changing renal Ca reabsorption and distal Ca load
*TRPM6*	9q21.13	607009	Hypomagnesemia with secondary hypocalcemia	AR	Severe hypomagnesemia, seizures in infancy, possible nephrocalcinosis	TRPM6 channel defect reduces intestinal and renal Mg^2+^ transport → profound hypomagnesemia and secondary effects on Ca handling
*OCRL*	Xq26.1	300535	Lowe syndrome/Dent disease 2	XLR	Proximal tubulopathy (Fanconi features), low-molecular-weight proteinuria, nephrocalcinosis; cataracts, neurodevelopmental features in Lowe syndrome	*OCRL* phosphatase defect disrupts endosomal trafficking and proximal tubule receptor recycling → tubular dysfunction and mineral handling abnormalities
*CYP24A1*	20q13.2	126065	Idiopathic infantile hypercalcemia 1	AR	Hypercalcemia, hypercalciuria, nephrocalcinosis; vitamin D hypersensitivity phenotype	Loss of vitamin D 24-hydroxylase reduces catabolism of active vitamin D → elevated 1,25(OH)_2_D and increased Ca absorption/excretion
*KCNJ1*	11q24.3	600359	Bartter syndrome type 2	AR	Antenatal/neonatal presentation with polyhydramnios, salt wasting, hypercalciuria, nephrocalcinosis	ROMK (*KCNJ1*) loss impairs K recycling in TAL → defective NKCC2 function and altered lumen voltage promoting Ca^2+^ loss
*PKHD1*	6p12.3-p12.2	606702	Autosomal recessive polycystic kidney disease (ARPKD)	AR	Collecting duct dilatation, early-onset cystic disease, progressive CKD; nephrocalcinosis may occur secondary to structural changes	Fibrocystin defect disrupts collecting duct morphogenesis and ciliary signaling → ductal dilation, stasis and predisposition to crystal retention
*PKD1*	16p13.3	601313	Autosomal dominant polycystic kidney disease (ADPKD)	AD	Progressive cystic disease, variable nephrocalcinosis; adult onset typical	Polycystin 1 dysfunction alters tubular architecture and flow dynamics, promoting stasis and secondary mineral deposition
*NPHP1*	2q13	256100	Nephronophthisis 1	AR	Tubulointerstitial nephropathy, polyuria, progressive CKD; nephrocalcinosis reported in some cases	Nephrocystin deficiency impairs ciliary function and tubular architecture, altering flow sensing and solute handling
*HNF1B*	17q12	137920	*HNF1B*-related disease (renal cysts and diabetes syndrome)	AD	Renal cysts, hypomagnesemia, electrolyte abnormalities, nephrocalcinosis in some patients; diabetes and genital tract malformations possible	Transcription factor defect disrupts nephron development and transporter expression, causing structural and transport abnormalities
*MT-ATP6*	mtDNA	590060	Mitochondrial *ATP6*-related disorders	Maternal Inheritance	Multisystem mitochondrial disease with tubulointerstitial nephropathy, variable nephrocalcinosis	Mitochondrial OXPHOS impairment reduces ATP for tubular transporters, increasing susceptibility to tubular injury and crystal retention
*CYP27B1*	12q14.1	609506	Vitamin D-dependent rickets type 1	AR	Rickets, biochemical abnormalities of vitamin D metabolism; nephrocalcinosis may occur with therapy or dysregulation	1α-hydroxylase defect alters active vitamin D synthesis and downstream calcium/phosphate balance

**Notes**: ↑, increase; →, leads to/results in.

## Data Availability

This manuscript is a narrative integrative review based exclusively on previously published literature. No new datasets were generated or analyzed during the current study; therefore, data sharing is not applicable.

## Abbreviations and Key Molecular Terms

The following abbreviations are used in this manuscript:

NGS	Next-Generation Sequencing: High-throughput DNA sequencing technology that allows for the simultaneous sequencing of millions of small DNA fragments.
WES	Whole Exome Sequencing: A genomic technique for sequencing all of the protein-coding regions (exones) of genes in a genome.
WGS	Whole Genome Sequencing: The process of determining the entirety, or nearly the entire, DNA sequence of an organism’s genome at a single time.
RNA	Ribonucleic Acid: A nucleic acid molecule essential in various biological roles in coding, decoding, regulation, and expression of genes.
VLBW	Very-Low-Birth-Weight: Birth weight < 1500 g; associated with increased metabolic, nutritional, and developmental vulnerability.
NC	Nephrocalcinosis: Deposition of calcium salts within renal parenchyma (medulla or cortex).
NL	Nephrolithiasis: Kidney stones; may coexist with NC in tubular disorders.
pH	Potential of Hydrogen: A logarithmic scale used to specify the acidity or basicity of an aqueous solution, defined as log[H^+^].
ATP	Adenosine Triphosphate: The primary energy carrier in all living organisms, capturing chemical energy obtained from the breakdown of food molecules.
MPTP	Mitochondrial Permeability Transition Pore: A protein pore in the inner mitochondrial membrane that, when open, leads to mitochondrial swelling and cell death.
ROS	Reactive Oxygen Species: Highly reactive chemical molecules formed due to the electron-accepting property of molecular oxygen, often causing oxidative stress.
NLRP3	NOD-Like Receptor Pyrin Domain Containing 3: An intracellular sensor that detects broad triggers to form an inflammasome, initiating an innate immune response.
TAL	Thick Ascending Limb: A segment of the loop of Henle in the kidney responsible for the active reabsorption of sodium, potassium, and chloride.
Ca^2+^	Calcium Ion: A vital divalent cation acting as a second messenger and structural component, strictly regulated in the blood and urine.
Mg^2+^	Magnesium Ion: A divalent cation that acts as a cofactor in hundreds of enzymatic reactions, including those involving ATP.
FHHNC	Familial Hypomagnesemia with Hypercalciuria and Nephrocalcinosis: A rare autosomal recessive tubular disorder characterized by renal magnesium and calcium wasting.
*CLDN19*	Claudin-19: A tight junction protein in the TAL of the loop of Henle; mutations impair the paracellular reabsorption of Mg^2+^ and Ca^2+.^
CaSR	Calcium-Sensing Receptor: A G protein-coupled receptor that senses extracellular calcium levels and regulates parathyroid hormone (PTH) secretion.
TRPV5	Transient Receptor Potential Vanilloid 5: An epithelial calcium channel in the distal tubule that serves as the gatekeeper for active renal calcium reabsorption.
NaPi-IIa	Sodium-Phosphate Cotransporter 2a: An apical membrane protein in the proximal tubule that mediates the majority of renal phosphate reabsorption.
NaPi-IIc	Sodium-Phosphate Cotransporter 2c: An electroneutral phosphate transporter in the proximal tubule, crucial for phosphate homeostasis in children.
*SLC34A1*	Solute Carrier Family 34 Member 1: The gene encoding the NaPi-IIa transporter; mutations lead to hypophosphatemic nephrolithiasis.
*SLC34A3*	Solute Carrier Family 34 Member 3: The gene encoding the NaPi-IIc transporter; mutations cause Hereditary Hypophosphatemic Rickets with Hypercalciuria.
*FGF23*	Fibroblast Growth Factor 23: A bone-derived hormone (phosphatonin) that inhibits phosphate reabsorption and suppresses vitamin D activation.
1,25(OH)_2_D	Calcitriol: The biologically active form of Vitamin D that increases intestinal absorption of calcium and phosphate.
IIH	Idiopathic Infantile Hypercalcemia: A disorder characterized by high blood calcium levels in infants, often due to mutations in *CYP24A1* or *SLC34A1*.
HHRH	Hereditary Hypophosphatemic Rickets with Hypercalciuria: A rare genetic disorder caused by *SLC34A3* mutations, resulting in renal phosphate-wasting and bone softening.
*GALNT3*	Polypeptide N-Acetylgalactosaminyltransferase 3: An enzyme responsible for the O-glycosylation of FGF23, protecting it from inactivation.
klotho–FGF	Klotho–Fibroblast Growth Factor Complex: The required receptor complex (comprising α-Klotho and FGFR1) that allows FGF23 to signal in the kidney.
*ATP6V1B1*	V-Type Proton ATPase B1 Subunit: An essential subunit of the proton pump that acidifies urine in the collecting duct; mutations cause dRTA.
*ATP6V0A4*	V-Type Proton ATPase a4 Subunit: A transmembrane subunit of the H^+^-ATPase pump; mutations are the most common genetic cause of distal RTA.
H^+^-ATPase	Hydrogen Adenosine Triphosphatase: A proton pump that uses ATP to transport hydrogen ions across membranes to regulate cellular and luminal pH.
dRTA	Distal Renal Tubular Acidosis: A condition where the distal tubule fails to secrete H^+^, leading to systemic metabolic acidosis and low blood potassium.
*SLC4A1*	Solute Carrier Family 4 Member 1: The gene encoding the Anion Exchanger 1 (AE1) protein in red blood cells and the kidney.
Cl^−^/HCO_3_^−^	Chloride/Bicarbonate Exchanger: A transport mechanism that maintains intracellular pH and acid–base balance by swapping these two ions across a membrane.
*AE1*	Anion Exchanger 1: A protein located on the basolateral membrane of alpha-intercalated cells that exports bicarbonate into the blood.
*OCRL*	Oculocerebrorenal Syndrome of Lowe: A gene encoding an inositol polyphosphate 5-phosphatase; mutations cause Lowe syndrome and Dent disease type 2.
*AGXT*	Alanine-Glyoxylate Aminotransferase: (Synonymous with AGT) The gene responsible for Primary Hyperoxaluria Type 1.
PH1	Primary Hyperoxaluria Type 1: The most severe form of primary hyperoxaluria, leading to kidney failure due to peroxisomal enzyme deficiency.
AGT	Alanine-Glyoxylate Aminotransferase: A liver-specific peroxisomal enzyme that converts glyoxylate to glycine, preventing the formation of oxalate.
RHPR	Recommended Health Promoting Resource (Context-dependent): Often refers to clinical guidelines, but in metabolic contexts, it may be a typo for GRHPR.
GRHPR	Glyoxylate and Hydroxypyruvate Reductase: An enzyme that reduces hydroxypyruvate to D-glycerate and glyoxylate to glycolate to maintain oxalate homeostasis.
PH2	Primary Hyperoxaluria Type 2: An autosomal recessive metabolic disorder caused by mutations in the *GRHPR* gene, leading to excessive oxalate production and kidney stones.
HOGA1	4-Hydroxy-2-Oxoglutarate Aldolase 1: A mitochondrial enzyme that catalyzes the final step of hydroxyproline metabolism; mutations cause Primary Hyperoxaluria Type 3.
PH3	Primary Hyperoxaluria Type 3: A metabolic disorder characterized by the inability to break down 4-hydroxy-2-oxoglutarate, resulting in calcium oxalate urolithiasis.
DNA	Deoxyribonucleic Acid: The double-stranded molecule that encodes the genetic instructions used in the development, functioning, and reproduction of all known organisms.
*HNF4A*	Hepatocyte Nuclear Factor 4 Alpha: A nuclear receptor transcription factor that regulates the expression of genes involved in lipid and glucose metabolism.
tRNA	Transfer Ribonucleic Acid: An adapter molecule composed of RNA that serves as the physical link between the mRNA and the amino acid sequence of proteins.
OXPHOS	Oxidative Phosphorylation: The metabolic pathway in mitochondria that uses energy released by the oxidation of nutrients to produce adenosine triphosphate (ATP).
NPHP	Nephronophthisis: A group of autosomal recessive cystic kidney diseases caused by defects in the primary cilia-basal body complex (ciliopathies).
BBS	Bardet-Biedl Syndrome: A pleiotropic genetic disorder involving the *BBSome* protein complex, which mediates vesicle transport to the primary cilium.
*HNF1B*	Hepatocyte Nuclear Factor 1 Beta: A transcription factor essential for the development of the kidneys, liver, and pancreas; mutations lead to renal cysts and diabetes.
*PKHD1*	Polycystic Kidney and Hepatic Disease 1: The gene encoding Fibrocystin, a large receptor protein involved in the structural and functional integrity of the primary cilium.
ARPKD	Autosomal Recessive Polycystic Kidney Disease: A severe genetic disorder typically presenting in infancy, characterized by bilateral cystic dilation of the renal collecting ducts.
*PKD1*	Polycystic Kidney Disease 1: The gene encoding Polycystin-1, a membrane protein that acts as a mechanosensor in the kidney tubule epithelium.
*PKD2*	Polycystic Kidney Disease 2: The gene encoding Polycystin-2, a calcium-permeable cation channel that interacts with Polycystin-1.
ADPKD	Autosomal Dominant Polycystic Kidney Disease: The most common inherited kidney disease, characterized by the progressive growth of numerous fluid-filled cysts.
*ATP6V1B1*	ATPase H^+^ Transporting V1 Subunit B1: A component of vacuolar ATPase (V-ATPase) responsible for acid-base homeostasis in the renal intercalated cells.
*ATP6V0A4*	ATPase H^+^ Transporting V0 Subunit A4: A subunit of the V-ATPase proton pump; mutations are a common cause of distal renal tubular acidosis.
*TRPV5*	Transient Receptor Potential Vanilloid 5: A highly selective calcium channel located in the apical membrane of the distal convoluted tubule, crucial for calcium reabsorption.
*FXYD2*	FXYD Domain Containing Ion Transport Regulator 2: The gamma subunit of the Na^+^/K^+^-ATPase that modulates the enzyme’s affinity for sodium and potassium ions.
Na^+^/K^+^-ATPase	Sodium-Potassium Adenosine Triphosphatase: An enzyme found in the plasma membrane of all animal cells that pumps sodium out and potassium into the cell against concentration gradients.
OFD1	Orofaciodigital Syndrome 1: A protein localized to the basal body of the primary cilium, essential for ciliogenesis and left-right symmetry during development.
IFT	Intraflagellar Transport: The bidirectional movement of protein complexes along the ciliary axoneme, powered by kinesin and dynein motors, necessary for ciliary assembly.

## References

[1] Spasiano A., Letavernier E., Ferraro P.M., Unwin R.J., Gambaro G. (2025). Kidney stone biology: Insights from genetics. Nephrol. Dial. Transplant..

[2] Oliveira B., Kleta R., Bockenhauer D., Walsh S.B. (2016). Genetic, pathophysiological, and clinical aspects of nephrocalcinosis. Am. J. Physiol. Ren. Physiol..

[3] Gefen A.M., Sethna C.B., Cil O., Perwad F., Schoettler M., Michael M., Angelo J.R., Safdar A., Amlie-Wolf L., Hunley T.E. (2023). Genetic testing in children with nephrolithiasis and nephrocalcinosis. Pediatr. Nephrol..

[4] Patterson J., Jacob Z., Reynolds B.C. (2022). Genetic evaluation of paediatric nephrocalcinosis: Phenotype-driven genetic panels reveal a rare diagnosis. Clin. Kidney J..

[5] Giovanella S., Ligabue G., Chester J., Magistroni R. (2023). Genomic Approaches for Monogenic Kidney Diseases: A Comparative Review of Diagnostic Methods and Precision Medicine Implications. Appl. Sci..

[6] Hillebrand P., Hoppe B. (2020). Plasma oxalate levels in primary hyperoxaluria type I show significant intra-individual variation and do not correlate with kidney function. Pediatr. Nephrol..

[7] Huang L., Qi C., Zhu G., Ding J., Yuan L., Sun J., He X., Wang X. (2022). Genetic testing enables a precision medicine approach for nephrolithiasis and nephrocalcinosis in pediatrics: A single-center cohort. Mol. Genet. Genom. MGG.

[8] Pavlovic N., Krizanac M., Kumric M., Vukojevic K., Bozic J. (2025). Mitochondrial Dysfunction: The Silent Catalyst of Kidney Disease Progression. Cells.

[9] Rusu E.E., Sorohan B.M., Pandele R., Popescu A., Bobeica R., Balanica S., Zilisteanu D.S., Iordache A., Lungu A., Ismail G. (2025). Phenotypes and the Importance of Genetic Analysis in Adult Patients with Nephrolithiasis and/or Nephrocalcinosis: A Single-Center Experience. Genes.

[10] Sayer J.A. (2017). Progress in Understanding the Genetics of Calcium-Containing Nephrolithiasis. J. Am. Soc. Nephrol. JASN.

[11] Gefen A.M., Zaritsky J.J. (2024). Review of childhood genetic nephrolithiasis and nephrocalcinosis. Front. Genet..

[12] Huang Y., Zhu W., Zhou J., Huang Q., Zeng G. (2024). Navigating the Evolving Landscape of Primary Hyperoxaluria: Traditional Management Defied by the Rise of Novel Molecular Drugs. Biomolecules.

[13] Martínez-Galindo R., Campuzano-Pérez M., Konstantouli A., Aguilar-Ramírez M.D.P., Rodríguez J.A.M., Abad-López P., Shabaka A., Cansino R. (2026). Clinical Approaches and Emerging Therapeutic Horizons in Primary Hyperoxaluria. J. Clin. Med..

[14] Giglio S., Montini G., Trepiccione F., Gambaro G., Emma F. (2021). Distal renal tubular acidosis: A systematic approach from diagnosis to treatment. J. Nephrol..

[15] Palazzo V., Provenzano A., Becherucci F., Sansavini G., Mazzinghi B., Orlandini V., Giunti L., Roperto R.M., Pantaleo M., Artuso R. (2017). The genetic and clinical spectrum of a large cohort of patients with distal renal tubular acidosis. Kidney Int..

[16] Godron A., Harambat J., Boccio V., Mensire A., May A., Rigothier C., Couzi L., Barrou B., Godin M., Chauveau D. (2012). Familial hypomagnesemia with hypercalciuria and nephrocalcinosis: Phenotype-genotype correlation and outcome in 32 patients with CLDN16 or CLDN19 mutations. Clin. J. Am. Soc. Nephrol. CJASN.

[17] Konrad M., Schlingmann K.P. (2014). Inherited disorders of renal hypomagnesaemia. Nephrol. Dial. Transplant..

[18] Hill F., Sayer J.A. (2019). Precision medicine in renal stone-formers. Urolithiasis.

[19] Dickson F.J., Sayer J.A. (2020). Nephrocalcinosis: A Review of Monogenic Causes and Insights They Provide into This Heterogeneous Condition. Int. J. Mol. Sci..

[20] Figueres L., Hourmant M., Lemoine S. (2020). Understanding and managing hypercalciuria in adults with nephrolithiasis: Keys for nephrologists. Nephrol. Dial. Transplant..

[21] Singh P., Harris P.C., Sas D.J., Lieske J.C. (2022). The genetics of kidney stone disease and nephrocalcinosis. Nat. Rev. Nephrol..

[22] Schell-Feith E.A., Kist-van Holthe J.E., van der Heijden A.J. (2010). Nephrocalcinosis in preterm neonates. Pediatr. Nephrol..

[23] Oh G.J., Butani L. (2024). Nephrocalcinosis in Neonates. NeoReviews.

[24] Pintus F., Giordano N., Giachino D.F., Mandrile G. (2025). Genetics of kidney stones and the role of genetic testing in prevention: A guide for urologists. Front. Med..

[25] Garunkstiene R., Levuliene R., Cekuolis A., Cerkauskiene R., Drazdiene N., Liubsys A. (2024). A Prospective Study of Nephrocalcinosis in Very Preterm Infants: Incidence, Risk Factors and Vitamin D Intake in the First Month. Medicina.

[26] Beal F., Patel A., Hulton S.A. (2024). Fifteen-minute consultation: An approach to the child with nephrocalcinosis. Arch. Dis. Child. Educ. Pract. Ed..

[27] Howles S.A., Thakker R.V. (2020). Genetics of kidney stone disease. Nat. Rev. Urol..

[28] Yoodee S., Peerapen P., Thongboonkerd V. (2024). Defining physicochemical properties of urinary proteins that determine their inhibitory activities against calcium oxalate kidney stone formation. Int. J. Biol. Macromol..

[29] Kaur M., Varanasi R., Nayak D., Tandon S., Agrawal V., Tandon C. (2025). Molecular insights into cell signaling pathways in kidney stone formation. Urolithiasis.

[30] Negri A.L., Spivacow F.R. (2023). Kidney stone matrix proteins: Role in stone formation. World J. Nephrol..

[31] Mulay S.R., Shi C., Ma X., Anders H.J. (2018). Novel Insights into Crystal-Induced Kidney Injury. Kidney Dis..

[32] Nazarian R., Lin N., Thaker S., Yang R., Wong G.C.L., Scotland K.B. (2025). What Causes Calcium Oxalate Kidney Stones to Form? An Update on Recent Advances. Uro.

[33] Downie M.L., Lopez Garcia S.C., Kleta R., Bockenhauer D. (2021). Inherited Tubulopathies of the Kidney: Insights from Genetics. Clin. J. Am. Soc. Nephrol. CJASN.

[34] Kuliasha C.A., Rodriguez D., Lovett A., Gower L.B. (2020). In situ flow cell platform for examining calcium oxalate and calcium phosphate crystallization on films of basement membrane extract in the presence of urinary ‘inhibitors’. CrystEngComm.

[35] Tamborino F., Cicchetti R., Mascitti M., Litterio G., Orsini A., Ferretti S., Basconi M., De Palma A., Ferro M., Marchioni M. (2024). Pathophysiology and Main Molecular Mechanisms of Urinary Stone Formation and Recurrence. Int. J. Mol. Sci..

[36] Berto S., Marangella M., De Stefano C., Milea D., Daniele P.G. (2021). Critical Reappraisal of Methods for Measuring Urine Saturation with Calcium Salts. Molecules.

[37] Hoogstraten C.A., Hoenderop J.G., de Baaij J.H.F. (2024). Mitochondrial Dysfunction in Kidney Tubulopathies. Annu. Rev. Physiol..

[38] Ying X., Chen Y., Hao Z., Liu H. (2025). The significance of reactive oxygen species in the formation of calcium oxalate stones and the protective effects of antioxidants on the kidneys. Front. Immunol..

[39] Gopalakrishnan J., Feistel K., Friedrich B.M., Grapin-Botton A., Jurisch-Yaksi N., Mass E., Mick D.U., Muller R.U., May-Simera H., Schermer B. (2023). Emerging principles of primary cilia dynamics in controlling tissue organization and function. EMBO J..

[40] Ryall R.L. (1997). Urinary inhibitors of calcium oxalate crystallization and their potential role in stone formation. World J. Urol..

[41] Prunotto M., Budd D.C., Meier M., Formentini I., Hartmann G., Pomposiello S., Moll S. (2012). From acute injury to chronic disease: Pathophysiological hypothesis of an epithelial/mesenchymal crosstalk alteration in CKD. Nephrol. Dial. Transplant..

[42] Huang R., Fu P., Ma L. (2023). Kidney fibrosis: From mechanisms to therapeutic medicines. Signal Transduct. Target. Ther..

[43] Spasiano A., Treccani M., De Tomi E., Malerba G., Gambaro G., Ferraro P.M. (2024). Characteristics and Yield of Modern Approaches for the Diagnosis of Genetic Causes of Kidney Stone Disease. Genes.

[44] Hildebrandt F. (2010). Genetic kidney diseases. Lancet.

[45] El-Achkar T.M., Eadon M.T., Kretzler M., Himmelfarb J., Kidney Precision Medicine P. (2024). Precision Medicine in Nephrology: An Integrative Framework of Multidimensional Data in the Kidney Precision Medicine Project. Am. J. Kidney Dis..

[46] Sun W., Han G., Fu C., Wang D., Wang S., Ding Z. (2025). Trace element signatures and regional differences in kidney stones: Insights from a high-prevalence region. Urolithiasis.

[47] Bergwitz C., Juppner H. (2010). Regulation of phosphate homeostasis by PTH, vitamin D, and FGF23. Annu. Rev. Med..

[48] Demoulin N., Aydin S., Gillion V., Morelle J., Jadoul M. (2022). Pathophysiology and Management of Hyperoxaluria and Oxalate Nephropathy: A Review. Am. J. Kidney Dis..

[49] Shee K., Stoller M.L. (2022). Perspectives in primary hyperoxaluria—Historical, current and future clinical interventions. Nat. Rev. Urol..

[50] Hoppe B. (2012). An update on primary hyperoxaluria. Nat. Rev. Nephrol..

[51] Karet F.E., Finberg K.E., Nelson R.D., Nayir A., Mocan H., Sanjad S.A., Rodriguez-Soriano J., Santos F., Cremers C.W., Di Pietro A. (1999). Mutations in the gene encoding B1 subunit of H^+^-ATPase cause renal tubular acidosis with sensorineural deafness. Nat. Genet..

[52] Alexander R.T., Gil-Pena H., Greenbaum L.A., Santos F., Adam M.P., Bick S., Mirzaa G.M., Pagon R.A., Wallace S.E., Amemiya A. (1993). Hereditary Distal Renal Tubular Acidosis. GeneReviews^®^.

[53] Konrad M., Schaller A., Seelow D., Pandey A.V., Waldegger S., Lesslauer A., Vitzthum H., Suzuki Y., Luk J.M., Becker C. (2006). Mutations in the tight-junction gene claudin 19 (CLDN19) are associated with renal magnesium wasting, renal failure, and severe ocular involvement. Am. J. Hum. Genet..

[54] Schlingmann K.P., Ruminska J., Kaufmann M., Dursun I., Patti M., Kranz B., Pronicka E., Ciara E., Akcay T., Bulus D. (2016). Autosomal-Recessive Mutations in SLC34A1 Encoding Sodium-Phosphate Cotransporter 2A Cause Idiopathic Infantile Hypercalcemia. J. Am. Soc. Nephrol. JASN.

[55] Fontecha-Barriuso M., Lopez-Diaz A.M., Guerrero-Mauvecin J., Miguel V., Ramos A.M., Sanchez-Nino M.D., Ruiz-Ortega M., Ortiz A., Sanz A.B. (2022). Tubular Mitochondrial Dysfunction, Oxidative Stress, and Progression of Chronic Kidney Disease. Antioxidants.

[56] Milliner D.S., Harris P.C., Sas D.J., Cogal A.G., Lieske J.C., Adam M.P., Bick S., Mirzaa G.M., Pagon R.A., Wallace S.E., Amemiya A. (1993). Primary Hyperoxaluria Type 1. GeneReviews^®^.

[57] Kim G.H. (2023). Primary cilia of the kidney: From ciliopathy to urinary concentration. Kidney Res. Clin. Pract..

[58] Hurd T.W., Hildebrandt F. (2011). Mechanisms of nephronophthisis and related ciliopathies. Nephron. Exp. Nephrol..

[59] Riddle H.A.L., Zhang S., Qian F., Williams J.C., Stubbs J.R., Rowe P.S.N., Parnell S.C. (2022). Kidney stone formation in a novel murine model of polycystic kidney disease. Am. J. Physiol. Ren. Physiol..

[60] Devuyst O., Knoers N.V., Remuzzi G., Schaefer F., Board of the Working Group for Inherited Kidney Diseases of the European Renal Association and European Dialysis and Transplant Association (2014). Rare inherited kidney diseases: Challenges, opportunities, and perspectives. Lancet.

[61] Khan S.R., Pearle M.S., Robertson W.G., Gambaro G., Canales B.K., Doizi S., Traxer O., Tiselius H.G. (2016). Kidney stones. Nat. Rev. Dis. Primers.

[62] Hou J., Rajagopal M., Yu A.S. (2013). Claudins and the kidney. Annu. Rev. Physiol..

[63] Weber S., Hoffmann K., Jeck N., Saar K., Boeswald M., Kuwertz-Broeking E., Meij I.I.C., Knoers N.V., Cochat P., Sulakova T. (2000). Familial hypomagnesaemia with hypercalciuria and nephrocalcinosis maps to chromosome 3q27 and is associated with mutations in the PCLN-1 gene. Eur. J. Hum. Genet. EJHG.

[64] Kausalya P.J., Amasheh S., Gunzel D., Wurps H., Muller D., Fromm M., Hunziker W. (2006). Disease-associated mutations affect intracellular traffic and paracellular Mg2+ transport function of Claudin-16. J. Clin. Investig..

[65] Riccardi D., Valenti G. (2016). Localization and function of the renal calcium-sensing receptor. Nat. Rev. Nephrol..

[66] Vezzoli G., Terranegra A., Rainone F., Arcidiacono T., Cozzolino M., Aloia A., Dogliotti E., Cusi D., Soldati L. (2011). Calcium-sensing receptor and calcium kidney stones. J. Transl. Med..

[67] Hannan F.M., Nesbit M.A., Zhang C., Cranston T., Curley A.J., Harding B., Fratter C., Rust N., Christie P.T., Turner J.J. (2012). Identification of 70 calcium-sensing receptor mutations in hyper- and hypo-calcaemic patients: Evidence for clustering of extracellular domain mutations at calcium-binding sites. Hum. Mol. Genet..

[68] Giudice M.L., Mihalik B., Dinnyes A., Kobolak J. (2019). The Nervous System Relevance of the Calcium Sensing Receptor in Health and Disease. Molecules.

[69] Lienhardt A., Bai M., Lagarde J.P., Rigaud M., Zhang Z., Jiang Y., Kottler M.L., Brown E.M., Garabedian M. (2001). Activating mutations of the calcium-sensing receptor: Management of hypocalcemia. J. Clin. Endocrinol. Metab..

[70] Prot-Bertoye C., Griveau C., Skjodt K., Cheval L., Brideau G., Lievre L., Ferriere E., Arbaretaz F., Garbin K., Zamani R. (2021). Differential localization patterns of claudin 10, 16, and 19 in human, mouse, and rat renal tubular epithelia. Am. J. Physiol. Ren. Physiol..

[71] Serna J., Bergwitz C. (2020). Importance of Dietary Phosphorus for Bone Metabolism and Healthy Aging. Nutrients.

[72] Cano-Marmol R.P., Ros-Madrid I., Andreo-Lopez M.C., Munoz-Torres M. (2025). Hypophosphatemia in the Diagnosis and Management of Primary Hyperparathyroidism. J. Clin. Med..

[73] Mura-Escorche G., Garcia-Suarez L.C., Lebredo-Alvarez I., Ramos-Trujillo E., Claverie-Martin F. (2025). Identification of a Novel Homozygous SLC34A1 Missense Mutation and a Heterozygous SLC34A3 Deletion in an Infant with Nephrocalcinosis, Failure to Thrive, and Hypercalcemia. Int. J. Mol. Sci..

[74] Prot-Bertoye C., Houillier P. (2020). Claudins in Renal Physiology and Pathology. Genes.

[75] Capelli S., Donghi V., Maruca K., Vezzoli G., Corbetta S., Brandi M.L., Mora S., Weber G. (2015). Clinical and molecular heterogeneity in a large series of patients with hypophosphatemic rickets. Bone.

[76] Pons-Belda O.D., Alonso-Alvarez M.A., Gonzalez-Rodriguez J.D., Mantecon-Fernandez L., Santos-Rodriguez F. (2023). Mineral Metabolism in Children: Interrelation between Vitamin D and FGF23. Int. J. Mol. Sci..

[77] Liu C.J., Cheng C.W., Tsai Y.S., Huang H.S. (2021). Crosstalk between Renal and Vascular Calcium Signaling: The Link between Nephrolithiasis and Vascular Calcification. Int. J. Mol. Sci..

[78] Zittermann A. (2025). Regulation of Renal and Extrarenal Calcitriol Synthesis and Its Clinical Implications. Int. J. Mol. Sci..

[79] Goretti Penido M., Alon U.S. (2012). Phosphate homeostasis and its role in bone health. Pediatr. Nephrol..

[80] Murray S.L., Wolf M. (2024). Calcium and Phosphate Disorders: Core Curriculum 2024. Am. J. Kidney Dis..

[81] Alamilla-Sanchez M., Alcalá Salgado M.A., Ulloa Galván V.M., Yanez Salguero V., Yamá Estrella M.B., Morales López E.F., Ramos García N.A., Carbajal Zárate M.O., Salazar Hurtado J.D., Delgado Pineda D.A. (2025). Understanding Renal Tubular Function: Key Mechanisms, Clinical Relevance, and Comprehensive Urine Assessment. Pathophysiology.

[82] Stehberger P.A., Schulz N., Finberg K.E., Karet F.E., Giebisch G., Lifton R.P., Geibel J.P., Wagner C.A. (2003). Localization and regulation of the ATP6V0A4 (a4) vacuolar H^+^-ATPase subunit defective in an inherited form of distal renal tubular acidosis. J. Am. Soc. Nephrol. JASN.

[83] Bao D., Wang Y., Zhao M.H. (2023). Oxalate Nephropathy and the Mechanism of Oxalate-Induced Kidney Injury. Kidney Dis..

[84] Emma F., Montini G., Parikh S.M., Salviati L. (2016). Mitochondrial dysfunction in inherited renal disease and acute kidney injury. Nat. Rev. Nephrol..

[85] Piko N., Bevc S., Hojs R., Ekart R. (2023). The Role of Oxidative Stress in Kidney Injury. Antioxidants.

[86] Marable S.S., Chung E., Park J.S. (2020). Hnf4a Is Required for the Development of Cdh6-Expressing Progenitors into Proximal Tubules in the Mouse Kidney. J. Am. Soc. Nephrol. JASN.

[87] Petropoulou A., Kypraios N., Rizopoulou D., Kouvela A., Maniatis A., Anastasopoulou K., Anastogianni A., Korfiatis T., Grafanaki K., Stamatopoulou V. (2025). Mitochondrial tRNA-Derived Diseases. Int. J. Mol. Sci..

[88] Satariano M., Ghose S., Raina R. (2024). The Pathophysiology of Inherited Renal Cystic Diseases. Genes.

[89] Gupta S., Ozimek-Kulik J.E., Phillips J.K. (2021). Nephronophthisis-Pathobiology and Molecular Pathogenesis of a Rare Kidney Genetic Disease. Genes.

[90] Iancu D., Ashton E. (2020). Inherited Renal Tubulopathies-Challenges and Controversies. Genes.

[91] Abou Alaiwi W.A., Lo S.T., Nauli S.M. (2009). Primary cilia: Highly sophisticated biological sensors. Sensors.

[92] Hartung E.A., Guay-Woodford L.M. (2014). Autosomal recessive polycystic kidney disease: A hepatorenal fibrocystic disorder with pleiotropic effects. Pediatrics.

[93] Alshriem L.A., Buqaileh R., Alorjani Q., AbouAlaiwi W. (2025). Ciliary Ion Channels in Polycystic Kidney Disease. Cells.

[94] Kim R., Kim T.M. (2026). The role of extracellular vesicles in kidney disease progression. Kidney Res. Clin. Pract..

[95] Buqaileh R., Alshriem L.A., AbouAlaiwi W. (2025). Ciliary G-Protein Coupled Receptor Signaling in Polycystic Kidney Disease. Int. J. Mol. Sci..

[96] Silverberg S.J., Walker M.D. (2024). Disorders of the Calcium-Sensing Receptor: Familial Hypocalciuric Hypercalcemia and Autosomal Dominant Hypocalcemia. UpToDate. https://www.uptodate.com/contents/disorders-of-the-calcium-sensing-receptor-familial-hypocalciuric-hypercalcemia-and-autosomal-dominant-hypocalcemia.

[97] Vall-Palomar M., Madariaga L., Ariceta G. (2021). Familial hypomagnesemia with hypercalciuria and nephrocalcinosis. Pediatr. Nephrol..

[98] Kiuchi Z., Nozu K., Yan K., Juppner H. (2023). Bartter Syndrome Type 1 Due to Novel SLC12A1 Mutations Associated With Pseudohypoparathyroidism Type II. JCEM Case Rep..

[99] Li J., Hu S., Nie Y., Wang R., Tan M., Li H., Zhu S. (2019). A novel compound heterozygous KCNJ1 gene mutation presenting as late-onset Bartter syndrome: Case report. Medicine.

[100] Bacchetta J., Lieske J.C. (2022). Primary hyperoxaluria type 1: Novel therapies at a glance. Clin. Kidney J..

[101] Gang X., Liu F., Mao J. (2022). Lumasiran for primary hyperoxaluria type 1: What we have learned?. Front. Pediatr..

[102] Gordon R.J., Li D., Doyle D., Zaritsky J., Levine M.A. (2020). Digenic Heterozygous Mutations in SLC34A3 and SLC34A1 Cause Dominant Hypophosphatemic Rickets with Hypercalciuria. J. Clin. Endocrinol. Metab..

[103] Dhir G., Li D., Hakonarson H., Levine M.A. (2017). Late-onset hereditary hypophosphatemic rickets with hypercalciuria (HHRH) due to mutation of SLC34A3/NPT2c. Bone.

[104] Cappellani D., Brancatella A., Morganti R., Borsari S., Baldinotti F., Caligo M.A., Kaufmann M., Jones G., Marcocci C., Cetani F. (2021). Hypercalcemia due to CYP24A1 mutations: A systematic descriptive review. Eur. J. Endocrinol..

[105] Leszczynska D., Szatko A., Latocha J., Kochman M., Duchnowska M., Wojcicka A., Misiorowski W., Zgliczyniski W., Glinicki P. (2024). Persistent hypercalcaemia associated with two pathogenic variants in the CYP24A1 gene and a parathyroid adenoma-a case report and review. Front. Endocrinol..

[106] Lewis R.A., Nussbaum R.L., Brewer E.D., Adam M.P., Bick S., Mirzaa G.M., Pagon R.A. (2019). Lowe Syndrome. GeneReviews^®^.

[107] De Matteis M.A., Staiano L., Emma F., Devuyst O. (2017). The 5-phosphatase OCRL in Lowe syndrome and Dent disease 2. Nat. Rev. Nephrol..

[108] Amaral S., Palha A., Bogalho P., Silva-Nunes J. (2023). Maturity-onset diabetes of the young secondary to HNF1B variants (HNF1B-MODY): A series of 10 patients from a single diabetes center. Diabetol. Metab. Syndr..

[109] Mateus J.C., Rivera C., O’Meara M., Valenzuela A., Lizcano F. (2020). Maturity-onset diabetes of the young type 5 a MULTISYSTEMIC disease: A CASE report of a novel mutation in the HNF1B gene and literature review. Clin. Diabetes Endocrinol..

[110] Reiterová J., Tesař V. (2022). Autosomal Dominant Polycystic Kidney Disease: From Pathophysiology of Cystogenesis to Advances in the Treatment. Int. J. Mol. Sci..

[111] Lopez-Garcia S.C., Emma F., Walsh S.B., Fila M., Hooman N., Zaniew M., Bertholet-Thomas A., Colussi G., Burgmaier K., Levtchenko E. (2019). Treatment and long-term outcome in primary distal renal tubular acidosis. Nephrol. Dial. Transplant..

[112] Cochat P., Hulton S.A., Acquaviva C., Danpure C.J., Daudon M., De Marchi M., Fargue S., Groothoff J., Harambat J., Hoppe B. (2012). Primary hyperoxaluria Type 1: Indications for screening and guidance for diagnosis and treatment. Nephrol. Dial. Transplant..

[113] Bockenhauer D., Jaureguiberry G. (2016). HNF1B-associated clinical phenotypes: The kidney and beyond. Pediatr. Nephrol..

[114] Stokman M., Lilien M., Knoers N., Adam M.P., Bick S., Mirzaa G.M., Pagon R.A., Wallace S.E., Amemiya A. (1993). Nephronophthisis-Related Ciliopathies. GeneReviews^®^.

[115] Hannan F.M., Kallay E., Chang W., Brandi M.L., Thakker R.V. (2019). The calcium-sensing receptor in physiology and in calcitropic and noncalcitropic diseases. Nat. Rev. Endocrinol..

[116] Sharma S., Place E., Lord K., Leroy B.P., Falk M.J., Pradhan M. (2016). Claudin 19-based familial hypomagnesemia with hypercalciuria and nephrocalcinosis in a sibling pair. Clin. Nephrol..

[117] Yuan M., Ma T., Fan Z., Li J., Zhang S. (2025). The calcium-sensing receptor: A comprehensive review on its role in calcium homeostasis and therapeutic implications. Am. J. Transl. Res..

[118] Marks J. (2019). The role of SLC34A2 in intestinal phosphate absorption and phosphate homeostasis. Pflug. Arch. Eur. J. Physiol..

[119] Claverie-Martin F., Vargas-Poussou R., Muller D., Garcia-Nieto V. (2015). Clinical utility gene card for: Familial hypomagnesemia with hypercalciuria and nephrocalcinosis with/without severe ocular involvement. Eur. J. Hum. Genet. EJHG.

[120] Sánchez-Cazorla E., Carrera N., García-González M.Á (2024). HNF1B Transcription Factor: Key Regulator in Renal Physiology and Pathogenesis. Int. J. Mol. Sci..

[121] Clissold R.L., Hamilton A.J., Hattersley A.T., Ellard S., Bingham C. (2015). HNF1B-associated renal and extra-renal disease-an expanding clinical spectrum. Nat. Rev. Nephrol..

[122] Cochat P., Fargue S., Bacchetta J., Bertholet-Thomas A., Sabot J.F., Harambat J. (2011). Primary hyperoxaluria. Nephrol. Ther..

